# Molecular Catalysts with Intramolecular Re–O Bond for Electrochemical Reduction of Carbon Dioxide

**DOI:** 10.1021/acs.inorgchem.0c01181

**Published:** 2020-08-17

**Authors:** Laura Rotundo, Dmitry E. Polyansky, Roberto Gobetto, David C. Grills, Etsuko Fujita, Carlo Nervi, Gerald F. Manbeck

**Affiliations:** † Chemistry Department, University of Torino, Via P. Giuria 7, 10125 Torino, Italy; ‡ CIRCC (Bari), University of Bari, Via Celso Ulpiani 27, 70126 Bari, Italy; § Chemistry Division, Brookhaven National Laboratory, Upton, New York 11973-5000, United States

## Abstract

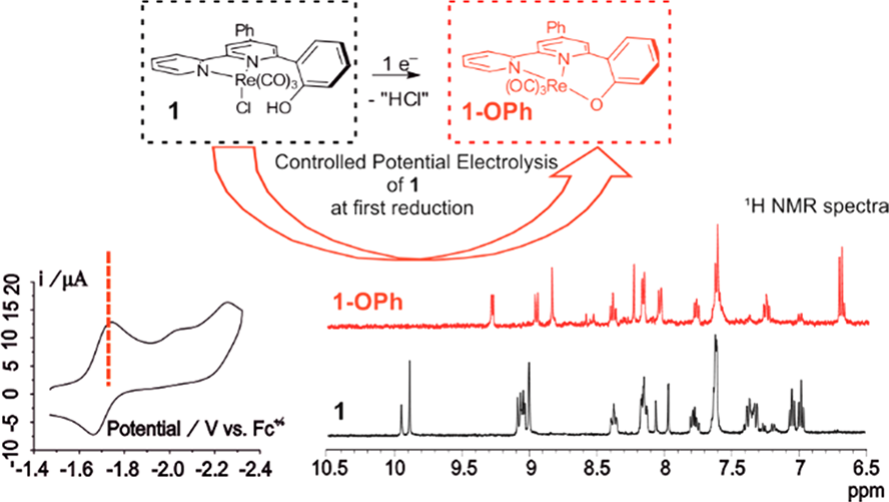

A new Re bipyridine-type complex, namely, *fac*-Re(pmbpy)(CO)_3_Cl (pmbpy = 4-phenyl-6-(2-hydroxy-phenyl)-2,2′-bipyridine), **1**, carrying a single OH moiety as local proton source, has been synthesized, and its electrochemical behavior under Ar and under CO_2_ has been characterized. Two isomers of **1**, namely, **1-***cis* characterized by the proximity of Cl to OH and **1-***trans*, are identified. The interconversion between **1-***cis* and **1-***trans* is clarified by DFT calculations, which reveal two transition states. The energetically lower pathway displays a non-negligible barrier of 75.5 kJ mol^–1^. The 1e^–^ electrochemical reduction of **1** affords the neutral intermediate **1-OPh**, formally derived by reductive deprotonation and loss of Cl^–^ from **1**. **1-OPh**, which exhibits an entropically favored intramolecular Re–O bond, has been isolated and characterized. The detailed electrochemical mechanism is demonstrated by combined chemical reactivity, spectroelectrochemistry, spectroscopic (IR and NMR), and computational (DFT) approaches. Comparison with previous Re and Mn derivatives carrying local proton sources highlights that the catalytic activity of Re complexes is more sensitive to the presence of local OH groups. Similar to **Re-2OH** (2OH = 4-phenyl-6-(phenyl-2,6-diol)-2,2′-bipyridine), **1** and **Mn-1OH** display a selective reduction of CO_2_ to CO. In the case of the Re bipyridine-type complex, the formation of a relatively stable Re–O bond and a preference for phenolate-based reactivity with CO_2_ slightly inhibit the electrocatalytic reduction of CO_2_ to CO, resulting in a low TON value of 9, even in the presence of phenol as a proton source.

## Abstract 2

A new Re bipyridine-type complex, namely, *fac*-Re(pmbpy)(CO)_3_Cl (pmbpy = 4-phenyl-6-(2-hydroxy-phenyl)-2,2′-bipyridine), **1**, carrying a single OH moiety as local proton source, has been synthesized, and its electrochemical behavior under Ar and under CO_2_ has been characterized. Two isomers of **1**, namely, **1-***cis* characterized by the proximity of Cl to OH and **1-***trans*, are identified.

## Introduction

**Scheme 1:**
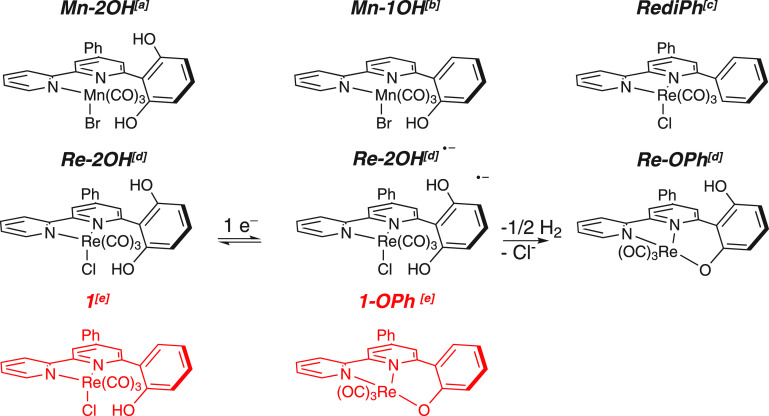
Sketches of Sample Mn and Re Bipyridine Electrocatalysts Bearing Local Proton Sources and Proposed Intermediates Formed during Electrochemical Reduction [a, refs (14 and 16); b, ref (45); c, ref (11); d, ref (13); e, This Work]

Carbon dioxide is a key greenhouse gas, and its concentration in the atmosphere is continuously increasing. The scientific community is drawing inspiration from natural photosynthesis in which CO_2_ and water are converted into glucose and O_2_ by various plants and algae just after capturing energy from sunlight. This process is an excellent model of direct chemical storage of solar energy. CO_2_ may be artificially converted into important chemical industry feedstocks such as CO or HCOOH and fuels like CH_3_OH and hydrocarbons (CH_4_, C_2_H_4_, C_2_H_6_).^1−6^ Another approach consists of converting solar light into electric energy and employing it for the electrochemical reduction of CO_2_.^7^ After the pioneer work of Lehn in the 1980s that first reported the capability of *fac*-Re(bpy)(CO)_3_Cl (bpy = 2,2′-bipyridine) in homogeneous solution to selectively reduce CO_2_ to CO,^8^ many new transition metal complexes were discovered.^5,9,10^ Among them, those derived from the original Rebpy and the corresponding Mn-bpy type received considerable interest^11−34^ due to performances and stability, especially when chemically bound to the electrode surface.^12,25,35−39^ The transition from the homogeneous to the heterogeneous approach, which in some cases displays superior stability and durability,^40^ can find considerable support whenever the intimate molecular mechanism responsible for the catalysis is initially deeply investigated in the homogeneous phase. The concept of a local proton source applied to the electrochemical reduction of CO_2_^41^ has been recently extended not only to the Mn-bpy system^14,16,23^ but also to the Rebpy complexes.^1,11,42−44^Scheme 1 shows a set of Mn and Re catalysts bearing pendent phenolic groups near the metal center acting as intramolecular proton sources. We initially reported *fac*-Mn(pdbpy)(CO)_3_Br^14,16^ (**Mn-2OH**), and recently we studied the effect of the two OH functionalities on *fac*-Re(pdbpy)(CO)_3_Cl^13^ (**Re-2OH**; pdbpy = 4-phenyl-6-(phenyl-2,6-diol)-2,2′-bipyridine). A similar catalyst, Mn(6-(2-hydroxyphenol)-2,2′-bipyridine)(CO)_3_Br (**Mn-1OH**) was previously described by Bocarsly and co-workers.^45^ We already concluded that the local proton source effects of the pdbpy ligand altered the selectivity for CO_2_ electroreduction depending on the metal involved.^13^ Indeed, while **Mn-2OH** gives the hydride form with subsequent production of formate, **Re-2OH** undergoes a different reduction pathway, and no formate is afforded. The intermediate **Re-OPh** (Scheme 1) is proposed to be produced after the first 1e^–^ reduction. **Re-OPh** is characterized by the presence of the intramolecular Re–O bond and undergoes electrochemical reduction to generate the actual catalyst in the CO_2_ conversion. IR-SEC measurements supported by DFT calculations suggested the formation of this intermediate following reductive deprotonation; however, its identity was not corroborated by other experimental techniques, and its precise effect on catalysis remained unclear.

The aims of the current work are to shed light on the fundamental aspects of this electrochemical mechanism and to provide additional spectroscopic evidence for phenolate coordination to the metal. In order to accomplish this goal, a novel complex bearing a single hydroxyl group instead of two, namely, *fac*-Re(pmbpy)(CO)_3_Cl (pmbpy = 4-phenyl-6-(2-hydroxy-phenyl)-2,2′-bipyridine), **1**, has been synthesized (Scheme 1). We hypothesized that a single pendent phenolic group could simplify the system by eliminating complications arising from the reductive or chemical deprotonation of the second phenolic group. Hydroxy-bipyridine-type ligands with the OH group close to the metal center are known to experience the electronic effects of the oxyanion formed by deprotonation to the catalytic site.^43^ Complex **1** has been characterized by cyclic voltammetry (CV), infrared spectroelectrochemistry (IR-SEC), and chemical reduction with Na–Hg. Data have been analyzed through comparison to Re(4,6-diphenyl-2,2′-bipyridine)(CO)_3_Cl (**RediPh**), which does not bear phenolic groups. The corresponding intermediate **1-OPh** has been isolated and characterized, and **1** has been tested as a catalyst for CO_2_ electroreduction with reactivity discussed in terms of the observed intermediates and compared to literature data for related complexes.

## Experimental Details

### General

All reagents for synthesis were purchased from Sigma-Aldrich or Alfa Aesar and used without further purification. Anhydrous toluene for synthesis was stored over activated molecular sieves under Ar. Acetonitrile for electrochemical experiments was dried on a solvent drying tower by passage through activated alumina then stored in a glovebox or transferred directly to electrochemical cells using standard Schlenk techniques. Alternatively, acetonitrile was freshly distilled over calcium hydride. The pmbpy ligand was synthesized by the Kröhnke reaction^46^ between the pyridinium salt [N-((2-pyridylacetyl) pyridinium iodide)]^47^ and [3-(2-methoxyphenyl)-1-phenylprop-2-en-1-one]^48^ (an α,β-unsaturated chalcone). NMR spectra were recorded on a 400 MHz Bruker Avance spectrometer (^1^H operating frequency 400 MHz) at 25 °C or, alternatively, on a JEOL ECP 400 FT-NMR spectrometer (^1^H operating frequency 400 MHz) at 25 °C. ^13^C spectra of **1** and **1-OPh** have been recorded on a JEOL ECZ 600 R. ^1^H and ^13^C chemical shifts are reported in parts per million relative to TMS (δ = 0) and referenced against solvent residual peaks. UV–vis spectra were measured on an Agilent 8453 spectrophotometer. Electrospray mass spectra (ESI-MS) were measured using a Thermo Scientific Q Exactive high resolution mass spectrometer. Samples **1** and **1-CH**_**3**_**CN**^**+**^ for microanalysis were dried in a vacuum to constant weight (20 °C, ca. 0.1 Torr). Elemental analysis (C, H, N) was performed in-house with a Fisons instrument 1108 CHNS-O Elemental Analyzer.

### Synthesis of 4-Phenyl-6-(methoxyphenyl)-2,2′-bipyridine

[N-((2-pyridylacetyl) pyridinium iodide)] (6.13 mmol), [3-(2-methoxyphenyl)-1-phenylprop-2-en-1-one] (6.13 mmol), ammonium acetate (63 mmol), and methanol (40 mL) were combined in a three-neck flask. The solution was purged with Ar and heated at reflux for 4 h. The progress of the reaction was monitored via TLC. After complete consumption of the reagents, the solvent was removed *invacuo* to afford a brown oil. The crude product was dissolved in ethyl acetate and washed three times with aqueous 10% NaHCO_3_. The oil collected after evaporation of ethyl acetate was purified on a silica gel column eluting with petroleum ether/ethyl acetate (7:1) to provide the title complex as a yellow oil in 56% yield. ^1^H NMR (400 MHz, (CD_3_)_2_SO): δ 8.75 (d, *J* = 4.7 Hz, 1H), 8.59 (d, *J* = 1.8 Hz, 1H), 8.53 (d, *J* = 7.9 Hz, 1H), 8.18 (d, *J* = 1.5 Hz, 1H), 7.95–8.02 (m, 2H), 7.89 (d, *J* = 8.5 Hz, 2H), 7.59 (t, *J* = 7.0 Hz, 2H), 7.46–7.55 (m, 2H), 7.22–7.29 (m, 2H), 7.16 (t, *J* = 7.6 Hz, 1H), 3.9 (s, 3H).

### Synthesis of 4-Phenyl-6-(2-hydroxyphenyl)-2,2′-bipyridine (pmbpy)

4-Phenyl-6-(methoxyphenyl)-2,2′-bipyridine (3 mmol) was dissolved in 30 mL of HBr (48% aqueous solution) and heated at reflux for 24 h. The mixture was then cooled to room temperature and the pH adjusted to pH 7 using a NaHCO_3_ aqueous solution. When the solution became neutral, pmbpy precipitated as a white solid, which was filtered and then washed with water and CH_2_Cl_2_. No further purification was needed (yield 89%). ^1^H NMR (400 MHz, (CD_3_)_2_SO): δ 8.82 (d, *J* = 4.4 Hz, 1H), 8.59 (s, 1H), 8.53 (s, 1H), 8.31 (t, *J* = 10.0 Hz, 2H), 8.1 (td, ^1^*J* = 6.2 Hz, ^2^*J* = 1.2 Hz, 1H), 8.05 (d, *J* = 7.0 Hz, 2H), 7.60 (m, 5H), 7.38 (td, ^1^*J* = 7.9 Hz, ^2^*J* = 1.16 Hz, 1H), 7.00 (d, *J* = 7.9 Hz, 1H).

### Synthesis of *fac*-Re(pmbpy)(CO)_3_Cl

Complex **1** was prepared according to the typical literature procedure.^13^ [Re(CO)_5_Cl] (0.500 mmol, 1 equiv) and the pmbpy ligand (0.501 mmol, 1.01 equiv) were dissolved in anhydrous toluene (20 mL) in a sealed flask and heated in a Biotage microwave reactor at a constant temperature of 130 °C for 1 h. After cooling of the reaction mixture to room temperature, petroleum ether was added to precipitate the yellow product, which was then centrifuged, filtered, and washed once with cold diethyl ether (yield: 92%). ^1^H NMR (400 MHz, (CD_3_)_2_SO): 9.96–9.89 s (1H), 9.03–9.08 (m, 2H), 9.00 (s, 1H), 8.37 (t, *J* = 8 Hz, 1H), 8.12–8.17 (m, 2H), 8.05 d–7.96 d (*J* = 1.8 Hz, 1H), 7.59–7.62 (m, 3H), 7.76–7.98 (m, 1H), 7.30–7.37 (m, 2H), 7.04 (t, *J* = 7.0 Hz, 1H), 6.97 (t, *J* = 7.5 Hz, 1H). ^13^C NMR (150 MHz, (CD_3_)_2_SO), carbonyl signals: 198.67, 198.50, 194.67, 193.85, 192.11, 190.78; 162.18 (Cq), 161.55 (Cq), 157.43 (Cq), 157.03 (Cq), 156.65 (Cq), 155.34 (Cq), 155.21 (Cq), 153.13 (−CH), 150.72 (Cq), 149.84 (Cq), 140.36 (−CH), 140.31 (−CH), 135.56 (Cq), 135.41 (Cq), 131.81 (−CH), 131.79 (−CH), 131.68(−CH), 131.33 (−CH), 131.25 (−CH), 131.19 (−CH), 129.91 (−CH), 129.84 (−CH), 129.43 (−CH), 129.13, 128.75 (−CH), 128.35 (−CH), 128.23 (−CH), 128.14 (−CH), 127.66 (−CH), 126.25 (−CH), 125.96 (−CH), 125.85 (−CH), 125.74 (−CH), 125.69 (−CH), 120.49 (−CH), 120.42 (−CH), 119.58 (−CH), 118.96 (−CH), 117.06 (−CH), 116.22 (−CH). UV–vis (CH_3_CN λ_max_): 379 nm. IR (CH_3_CN, ν̃_CO_/cm^–1^): 2022, 1919, 1895 cm^–1^. HRMS (ESI+) *m*/*z* calcd for [M – Cl + Na]^+^: 653.0254. Found: 653.0223. [M – Cl + CH_3_CN]^+^: 636.0933. Found: 636.0910. [M–OH]^+^: 614.0407. Found: 614.0620. Anal. Calcd (%) for C_25_H_16_ClN_2_O_4_Re: C (47.66), H (2.56), N (4.45). Found: C (47.02), H (3.2), N (4.45).

### Synthesis of *fac*-[Re(pmbpy)(CO)_3_(CH_3_CN)](PF_6_)

The acetonitrile complex was prepared by heating complex **1** (7 mmol, 1 equiv) and AgPF_6_ (7 mmol, 1 equiv) in anhydrous acetonitrile (25 mL) at reflux overnight. After cooling of the mixture, it was filtered through Celite to remove AgCl, and the solution was then evaporated to dryness (yield: 60%). ^1^H NMR (400 MHz, CD_3_CN): 9.08 d (*J* = 5.1 Hz, 1H), 8.70–8.72 m (2H), 8.32 t (*J* = 7.8 Hz, 1H), 8.03 d (*J* = 2.0 Hz, 1H), 7.97–7.99 m (2H), 7.73 td (^1^*J* = 5.4 Hz, ^2^*J* = 2.5 Hz, 1H), 7.60–7.63 m (2H), 7.54 d (*J* = 3.2 Hz, 1H), 7.45 t (*J* = 7.3 Hz, 1H), 7.35 d (*J* = 7.6 Hz, 1H), 7.05–7.12 m (2H). ^13^C NMR (100 MHz, CD_3_CN) carbonyl signals: 194.09, 189.85, 184.17; 157.34 (Cq), 156.84 (Cq), 154.36 (Cq), 153.92 (−CH), 152.07 (Cq), 151.71 (Cq), 140.63 (−CH), 135.28 (Cq), 132.03 (−CH), 131.13 (−CH), 130.32 (−CH), 129.59 (−CH), 127.84 (−CH), 126.85 (−CH), 125.14 (−CH), 122.46 (Cq), 120.81 (−CH), 120.36 (−CH), 120.13 (−CH), 117.45 (−CH), 116.68 (−CH), 116.27 (−CH). (Figures S9 and S10). Anal. Calcd (%) for C_27_H_19_F_6_N_3_O_4_PRe: C (41.54), H (2.45), N (5.38). Found: C (40.86), H (3.16) N (5.22). IR (CH_3_CN, ν_CO_/cm^–1^): 2039, 1942, 1927 cm^–1^ (Figure S19).

### Synthesis of **1-OPh**

In two-compartment electrochemical cell inside an N_2_-filled glovebox, a 0.5 mM solution of **1** in CH_3_CN containing 0.035 mM Bu_4_NPF_6_ was subject to controlled potential electrolysis at −1.7 V vs Fc^+/0^ using a Pt mesh electrode. After complete consumption of **1** as judged by CV, the solution was evaporated to dryness. The resultant solid was dissolved in *d*_6_-DMSO and analyzed by NMR in a J-Young tube. ^1^H NMR (400 MHz, (CD_3_)_2_SO): 9.28 d (*J* = 5.1 Hz, 1H), 8.96 d (*J* = 8.6 Hz, 1H), 8.84 s (1H), 8.39 t (*J* = 8.2 Hz, 1H), 8.24 s (1H), 8.17 d (*J* = 8.1 Hz, 2H), 8.05 d (*J* = 8.1 Hz, 1H), 7.77 t (*J* = 6.7 Hz, 1H), 7.57–7.64 m (3H), 7.26 t (*J* = 7.5 Hz, 1H), 6.71 d, (*J* = 7.5 Hz, 2H). ^13^C NMR (150 MHz, (CD_3_)_2_SO): 199.50, 197.80, 193.68, 171.64 (Cq), 157.87 (Cq), 157.70 (Cq), 155.41 (Cq), 153.64 (Cq), 151.53 (−CH), 140.44 (−CH), 136.05 (Cq), 133.56 (−CH), 131.05 (−CH), 130.34 (−CH), 129.72 (−CH), 129.44 (−CH),126.75 (−CH), 124.83 (−CH), 120.87 (−CH), 120.27 (Cq), 119.55 (−CH), 119.45 (−CH), 118.50 (−CH). IR (CH_3_CN, ν_CO_/cm^–1^): 2013, 1907, 1884 cm^–1^.

### Electrochemistry

Cyclic voltammetry (CV) and controlled potential electrolysis (CPE) experiments were performed using a BASi Epsilon potentiostat. Data were collected in dry acetonitrile with tetrabutylammonium hexafluorophosphate Bu_4_NPF_6_ (0.1 M) as the supporting electrolyte. CVs were collected in a single-compartment cell with a three-electrode configuration using a glassy carbon (GC) working electrode (*d* = 3 mm), a Pt counter electrode, and a solid-state Ag/AgCl reference electrode (eDAQ) or a silver wire pseudo-reference. CVs at 195 K were collected in a sealed cell submerged in a dry ice/acetone bath. All potentials are reported relative to the ferrocenium/ferrocene couple (Fc^+/0^). Solutions were saturated with Ar or CO_2_ that were passed through an acetonitrile prebubbler to maintain a constant concentration of analyte. CPE experiments under N_2_ or CO_2_ were performed in two-compartment cells where anodic and cathodic compartments were separated by a glass frit. Preparative electrolysis experiments were carried out in an N_2_-filled glovebox, and Pt mesh was used as the working electrode or counter-electrode. CPE under CO_2_ was performed in a 30 mL cell using a glassy carbon rod working electrode and an aqueous SCE reference electrode in the cathodic compartment and a Pt wire counter electrode in the second compartment. Some experiments under CO_2_ were performed in the presence of water or methanol (5% by volume). A controlled flow of CO_2_ (50 mL min^–1^), measured just before arrival into the cell, was maintained during the CPE measurements by means of a Smart Trak 100 (Sierra) flow controller. The cell was airtight and equipped with a bubbler that maintained the inner atmosphere but avoided gas overpressure. Quantitative analysis of CO_2_ reduction products was carried out as reported previously.^11^

### Spectroelectrochemistry

A custom reflective infrared spectroelectrochemical (IR-SEC) cell equipped with boron-doped diamond working, platinum counter, and solid-state leakless miniature Ag/AgCl reference (EDAQ) electrodes was attached to a Fourier transform infrared (FTIR) spectrometer (Bruker, IFS 66/S) using a VeeMAX III Variable Angle Specular Reflectance Accessory (Pike Technologies), and the IR absorption changes were monitored following application of fixed potentials.

### Na–Hg Reduction

Sodium amalgam reduction was performed in a homemade vessel equipped with a quartz spectrophotometric cell separated by a glass frit from a second compartment containing 0.5% Na in Hg. Samples were prepared under a high vacuum with vacuum distilled CH_3_CN and were reduced gradually by introducing small portions to the amalgam chamber under a vacuum.

### DFT Calculations

Gaussian 16, Revision B.01 and Gaussian 09, Revision D.01 packages^49^ were adopted for all DFT calculations. Solvent effects were taken into account by the conductor-like polarizable continuum model (CPCM)^50,51^ with acetonitrile as a solvent. No constraints were imposed during geometry optimizations. The B3LYP functional,^52,53^ with the optimized def2-TZVP basis set for Re and Cl and the def2-SVP basis set^54,55^ for all other atoms was employed. The D3 version of Grimme’s dispersion method was applied adopting the Becke–Johnson damping scheme.^56^ Gibbs free energies were determined using thermal corrections for entropy and enthalpy at 298 K to the electronic energies. In these calculations, the computed harmonic frequencies were scaled by 0.965 to account for anharmonicity. For radical anions, the unrestricted Kohn–Sham formalism was adopted. The nature of all stationary points was confirmed by normal-mode analysis.

## Results and Discussion

### Synthesis and Characterization

The pmbpy ligand was prepared by the Kröhnke reaction similarly to pdbpy by using appropriately modified chalcone and pyridinium salts as precursors to cyclization.^16^ In this work, higher yields were obtained by introducing the methoxy precursor to the pmbpy ligand as a part of the chalcone. Complex **1** was obtained as a pure yellow solid from the reaction of Re(CO)_5_Cl with the pmbpy ligand in toluene. The infrared (IR) spectrum confirms the *fac* geometry with three strong CO stretching frequencies at 2022, 1919, and 1895 cm^–1^.

**Figure 1:**
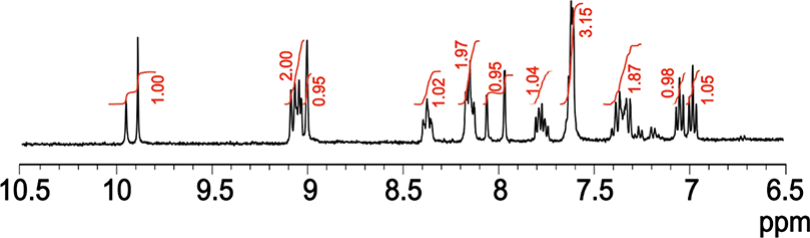
^1^H NMR spectrum of [Re(pmbpy)(CO)_3_Cl] (**1**) in *d*_6_-DMSO.

The 298 K ^1^H NMR spectrum of **1** is shown in Figure 1, and NMR correlation spectroscopy (COSY) with all signal assignments and the ^13^C spectrum are provided as Supporting Information (Figures S1–S4). Notably, there are two sharp resonances of unequal intensity at 9.96 and 9.89 ppm assigned to the −O*H* proton. The total integration of these two peaks is 1, and they are present in the same ratio for each preparation of the complex. The −O*H* resonances are shifted downfield relative to the two resonances of **Re-2OH** at 9.60 and 9.56 ppm,^13^ but the similar separation suggests two environments differing by comparable electronic effects. Additional evidence for the proposed isomers is found in separate resonances of the H_5_ singlets that appear in comparable ratios to the −O*H* singlets (Figure S1). The carbonyl region of the ^13^C NMR spectrum of **1** reveals six carbonyl resonances instead of three in agreement with the presence of two isomers, and two sets of resonances of several other carbon signals are also evident (Figure S3).

**Figure 2:**
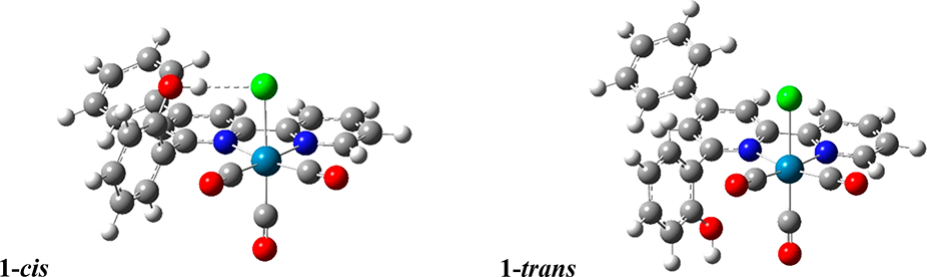
Representation of the two isomers **1**-*cis* and **1-***trans* computed by DFT calculations.

The two possible isomeric structures were investigated by DFT calculations (Figure 2). The form **1-***cis* is the most stable, by 5.1 kJ mol^–1^, due to the interaction between the −OH group and the Cl atom.

Intrigued by the NMR that evidenced a non-negligible rotational barrier of the phenolic group, we searched for two transition states connecting the two isomers. The first, ^**TS**^**1′**, may be thought to be obtained by the counterclockwise rotation of the **1-***cis* phenolic group (i.e., the OH group is on the opposite side of the Re(CO)_3_ moiety, see Figure 2), while the second, ^**TS**^**1′′**, may be thought to be reached by the clockwise rotation of the **1-***cis* phenolic group (i.e., the OH group is on the same side of the Re(CO)_3_ moiety). As expected, the energy barrier of ^**TS**^**1′** (75.5 kJ mol^–1^, single negative frequency at −28.8 cm^–1^) is lower than that of ^**TS**^**1′′** (95.0 kJ mol^–1^, single negative frequency at −35.0 cm^–1^), because the rotation of the phenolic group occurs when the OH moiety is far from Re(CO)_3_. The relatively high rotational barrier accounts for the presence of the two isomers and hence the splitting of NMR signals observed experimentally.

### Electrochemistry

**Figure 3:**
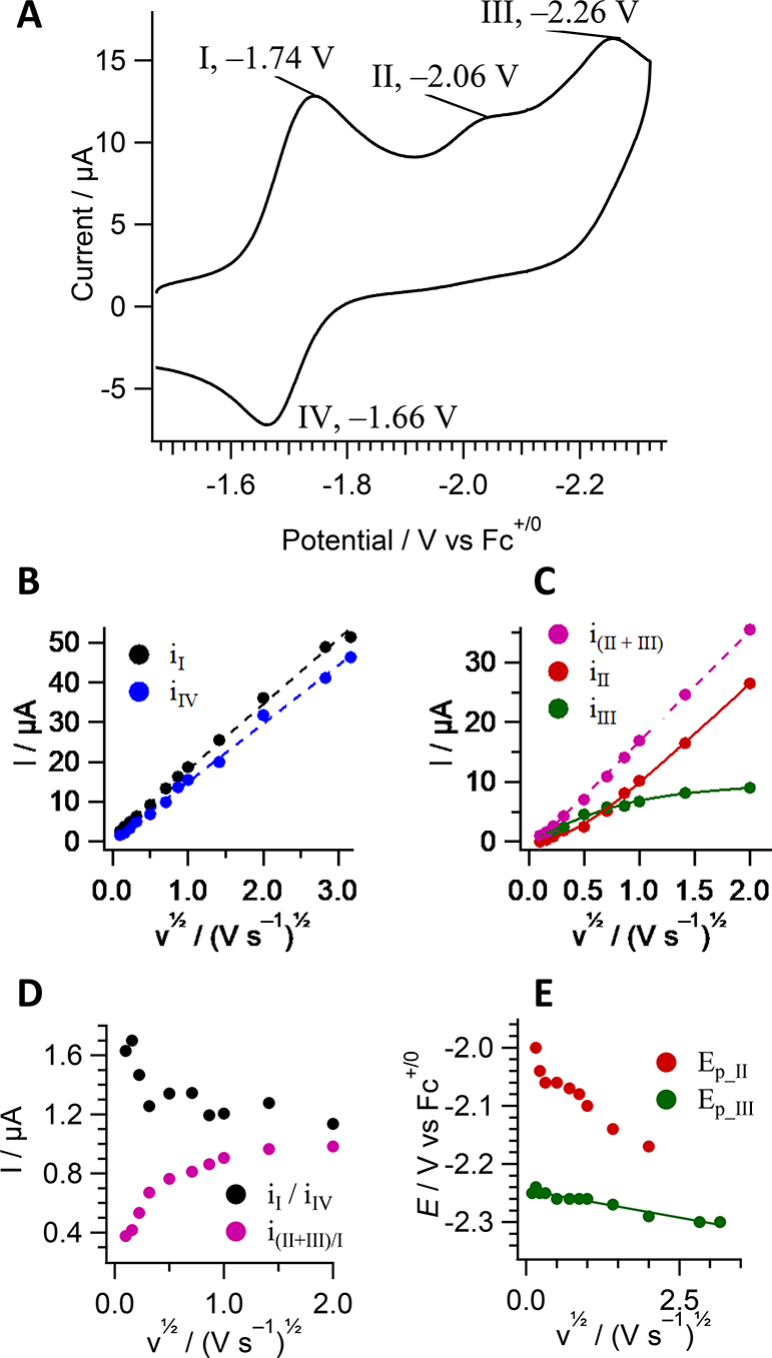
(A) Cyclic voltammogram of 0.4 mM **1** in Ar-saturated CH_3_CN with 0.1 M Bu_4_NPF_6_ at a glassy carbon electrode, scan rate = 0.25 V s^–1^. (B) Current at peaks I and IV. (C) Current at peaks II and III. (D) Ratios of currents at peaks I/IV and the sum of (II + III)/I. (E) Peak potentials of reductions II and III.

The cyclic voltammogram (CV) of **1** in anhydrous acetonitrile exhibits three reduction processes labeled I–III at intermediate sweep rates, ν (0.05 to 2 V s^–1^; Figure 3). Reduction I is reversible with an associated reoxidation wave IV and *E*_1/2_ = −1.70 vs Fc^+/0^. At a scan rate of 0.25 V s^–1^ wave II is irreversible and wave III is quasireversible, with peak potentials at −2.06 and −2.26 V, respectively.

Peak currents at waves I and IV are linearly dependent upon ν^1/2^ consistent with diffusional processes (Figure 3B), but the ratio of *i*_I_/*i*_IV_ decreases from a maximum of ∼1.6 at slow scan rates to ∼1 as the sweep rate is increased (Figure 3D). The decreasing current ratio *i*_I_/*i*_IV_ could be a simple effect of different diffusion coefficients of the oxidized and reduced species of the I/IV couple; however, further observations below suggest the participation of a chemical step and an additional reduction near wave I.

Peak currents at waves II and III are shown in Figure 3C, and sample CV’s with variable scan rates are shown in Figure S11. At the lowest limit of 0.01 V s^–1^, wave II is absent. As the scan rate is increased, the current at wave II increases at the expense of the current at wave III, and wave II shifts in potential from about −2 V to −2.15 V, after which it begins to overlap with wave III. The sum of peak currents at waves II and III remains proportional to ν^1/2^ throughout the entire range of sweep rates. The ratio of currents at (peaks II + III)/peak I is ∼0.5 at 0.05 mV s^–1^ and increases to ∼1 at 4 V s^–1^. Above 4 V s^–1^, it is difficult to differentiate currents from overlapping processes II and III.

Multisweep voltammograms (Figure S12) show decreasing current for reduction I and a complete loss of reduction II without accumulation of any distinct redox processes. For comparison, the multisweep CV of [Re(4,6-diphenyl-2,2′-bpy)(CO)_3_Cl]^11^ is also shown. This complex differs only by the absence of the −OH group and represents the electrochemical behavior expected of [Re(bpy)(CO)_3_Cl]-type complexes. The dominant reductive pathway follows an EEC mechanism with a reversible [Re(bpy)(CO)_3_Cl]^0/–^ couple and an irreversible [Re(bpy)(CO)_3_Cl]^−/2–^ couple followed by chloride dissociation to produce [Re(bpy)(CO)_3_]^−^,^15^ although a competing ECE pathway with a relatively slow dissociation of the chloride from the singly reduced radical is possible as well.^57^ Dimerization of the singly reduced species is possible,^57,58^ but we did not observe characteristic features of dimerization during reduction or reoxidation, especially at a 0.4 mM analyte concentration. Multiple scans of the [Re(4,6-diphenyl-2,2′-bpy)(CO)_3_Cl]^0/–^ couple (Figure S12B) verified its reversibility while multiple scans through the second reduction showed the accumulation of the solvento complex indicated by the appearance of the reversible [Re(4,6-diphenyl-2,2′-bpy)(CO)_3_(CH_3_CN)]^+/0^ couple, which typically appears at less cathodic potentials than the reduction of the chloro complex.^11,59^ CVs of **1** measured at 195 K showed only the typical EEC mechanism (Figure S13).

**Scheme 2:**
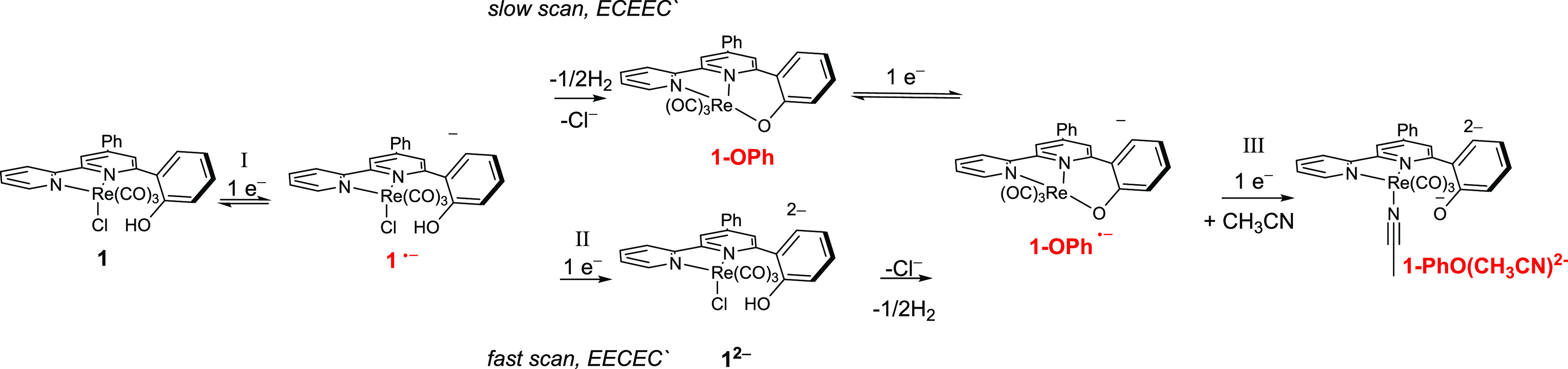
Proposed Electrochemical Mechanisms for Reduction of **1** (Species Observed by IR-SEC Labeled in Red)

Collectively, these observations indicate reductive mechanisms differing from the usual EEC pathway. Scan-rate-dependent currents at waves I, II, and III suggest the involvement of one or more chemical steps proposed in Scheme 2, and evidence for the assignments are provided by CV (Figures 3, S11–S13), IR-SEC (Figures 5, 6, S18, S21), chemical reduction with sodium amalgam (Figure S17), and chemical deprotonation of **1** (Figure S16). The potential of the first reduction and its reversible nature are similar to related complexes,^11,59,60^ allowing assignment of the I/IV couple as [Re(pmbpy)(CO)_3_Cl]^0/–^ (or **1**^0/–^). Subsequent competing mechanisms can be isolated at the fast and slow scan rate extremes and assignments made by comparison to [Re(4,6-diphenyl-2,2′-bpy)(CO)_3_Cl] (**RediPh)**. At 1 V s^–1^, peak II is well-resolved from peak III, and the current ratio *i*_II_/*i*_I_ of ∼0.5 is similar to the ratio observed for the irreversible **RediPh^–/2–^** reduction at −2.1 V vs Fc^+/0^. This process in the reference compound also shifts positive by ∼100 mV as the scan rate is decreased 100 mV s^–1^ due to its irreversible nature. Peak III is over 150 mV more negative than the **RediPh^–/2–^** reduction, ruling out an analogous assignment to peak III. Therefore, the irreversible peak II is assigned to the reduction of [Re(pmbpy)(CO)_3_Cl]^−^ followed by fast chloride dissociation to produce [1 **− Cl**^**–**^]**^–^** (Scheme 2) by the standard EEC mechanism through wave II.

The diminished CV current of peak II as the scan rate is slowed suggests that a chemical reaction of modest rate consumes the singly reduced complex, **1**^•–^, on the time scale of the CV experiment (Figure S11). At 10 mV s^–1^, peak II is absent showing complete consumption of **1**^•–^ by the alternate pathway. The nature of this pathway is proposed as an ECE mechanism in which the chemical step is a composite net H atom loss (reductive deprotonation) and chloride dissociation to produce the phenolate-ligated **1-OPh**, and characterization of this species will be presented below. As a neutral Re(I) complex with an anionic ligand, **1-OPh**, will have similar electronic structure and reduction potential to **1**, therefore the high *i*_I_/*i*_IV_ current ratio (Figure 3D) at slow scan rate is attributed to further reduction to **1-OPh**^•–^ after the chemical step.

At fast or slow scan rates, peak III is present without significant changes in potential or current. Its assignment is proposed as the **1-OPh**^**–/2–**^ couple, which is a shared final electron transfer step on both pathways, namely, EEC**E***C***′** and ECE**E***C***′**. The shared chemical step indicates that both mechanisms provide **1-OPh**^•–^ prior to the final step with the difference being whether the Cl^–^ dissociation and H atom loss occur after one or two initial reductions of the complex. The **1-OPh**^**–/2–**^ couple is most accurately described as an EC process itself because dissociation of the phenolate is proposed (see below).

**Figure 4:**
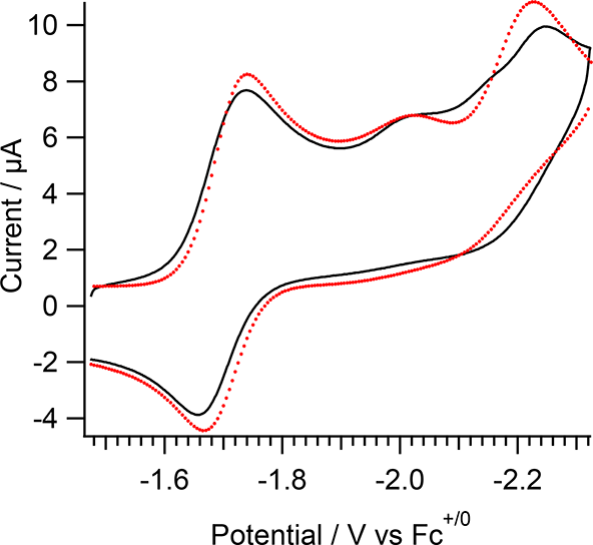
Experimental (black) and simulated (red) reductive voltammograms of **1** according to the mechanism in Scheme 2 with *k*_Ca_ = 920 s^–1^ for the EE*Ca*EC′ pathway and *k*_Cb_ = 0.43 s^–1^ for the E*Cb*EEC′ pathway. See Figure S14 for additional details.

The proposed reductive mechanism was simulated using Digisim^61^ software, and parameters were adjusted until reasonable models of the data collected at 100 and 1000 mV s^–1^ were produced (Figure 4 and S14). A perfect simulation was not expected for several reasons. The initial state of **1** exists as two isomers with potentially different rates of chemical steps. The low solubility of **1** may cause a more pronounced effect of a slightly sloped baseline since the software models capacitance as a constant value. Finally, the chemical steps of chloride dissociation, net H atom loss (1/2 H_2_), and phenolate coordination are modeled as a single step with equilibrium *K*_eq_ and a rate determining “*k*”, while in reality heterogeneous or homogeneous electron transfers of any putative intermediates are possible and may contribute to wave distortions. Nevertheless, the model shown in Figure 4 captures the experimental properties well, and the EEC portion of the fast scan pathway in Scheme 2 (*k*_Ca_ = 920 s^–1^) was simulated in good agreement with the reference compound [(4,6-diphenyl-2,2′-bipyridine)Re(CO)_3_Cl] (Figure S15) for which *k*_f_Cl-loss_ = 900 s^–1^.

The simulated mechanism reproduces the scan rate dependent potential and current of peak II. The potential shifts due to the irreversible reaction that drives the equilibrium toward the product, and the current is low at slow scan rates due to the competing ECEEC′ pathway, with *k*_Cb_ = 0.43 s^–1^. Both chemical steps represent the same processes occurring at species differing by an additional reduction, and the extra charge accelerates the reaction rate by over 2000-fold. The simulated mechanism shows that *E*_1/2_ for the reduction of **1**-**OPh** along the ECEEC′ pathway is only 30 mV more negative than that of **1**. The implications of these comparable potentials will be discussed in the context of spectroelectrochemistry and preparative electrolysis.

### Infrared Spectroelectrochemistry (IR-SEC)

The reductive electrochemical mechanism was investigated by infrared spectroelectrochemistry (IR-SEC) in N_2_-saturated CD_3_CN where the improved solvent window relative to CH_3_CN allows observation of the changes in carbonyl stretching frequencies as well as low energy bands associated with aromatic vibrational modes.

**Figure 5:**
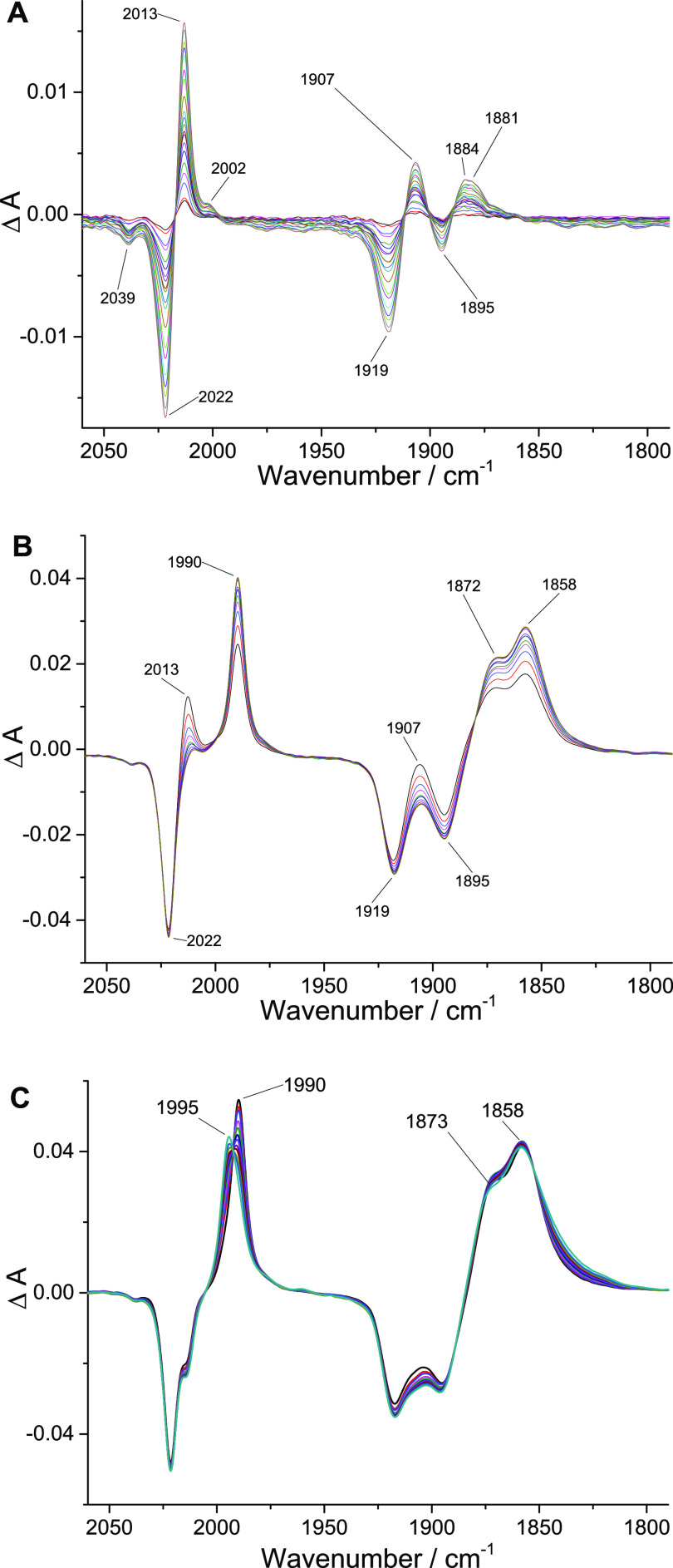
Difference IR spectra (expanded metal carbonyl region) measured during controlled potential electrolysis of **1** in CD_3_CN containing 0.1 M Bu_4_NPF_6_ under a N_2_ atmosphere. Applied potentials: (A) −1.5 V vs Fc^+/0^; (B) −1.7 V vs Fc^+/0^; and (C) −2.3 V vs Fc^+/0^.

**Figure 6:**
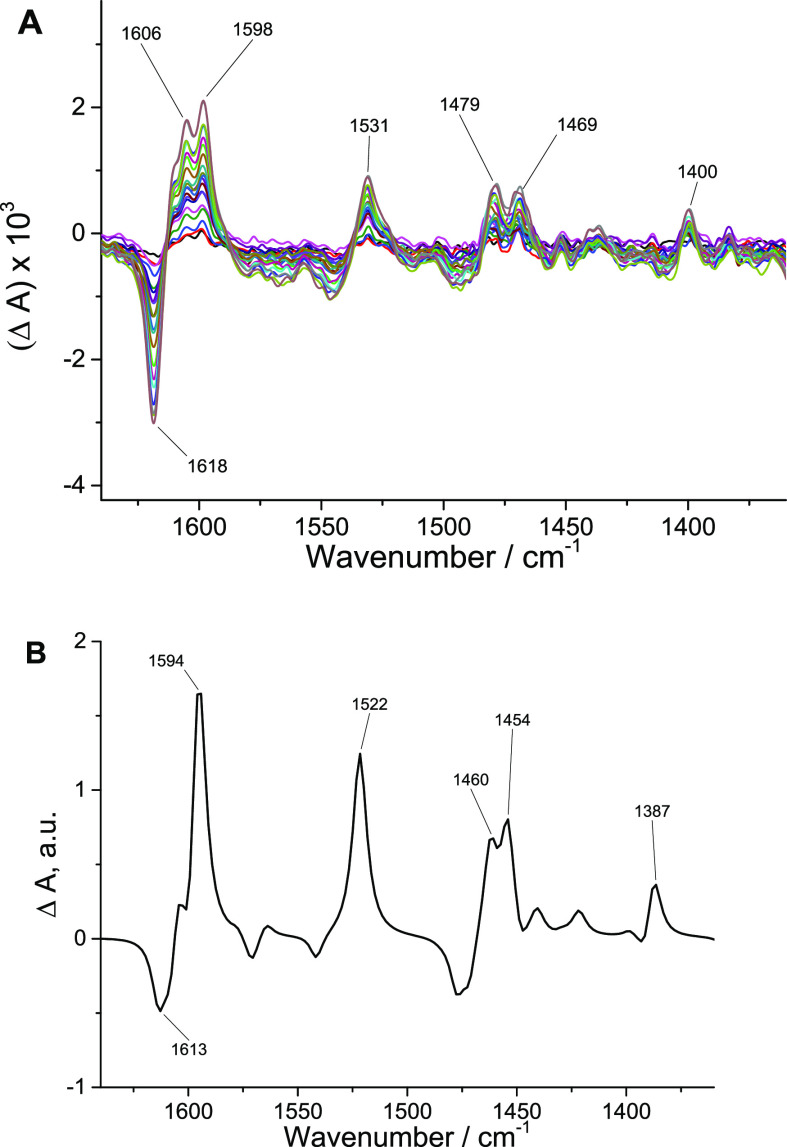
(A) Difference IR spectra (expanded ligand region) measured during controlled potential electrolysis of **1** at −1.5 V vs Fc^+/0^ in CD_3_CN containing 0.1 M Bu_4_NPF_6_ under a N_2_ atmosphere. (B) Difference IR spectrum obtained from subtraction of DFT calculated spectrum of **1**-**OPh** from calculated spectrum of **1**. Wavenumber axis is scaled by a factor of 0.965.

Electrolysis at the onset of the first reduction (−1.5 V vs Fc^+/0^) resulted in an immediate bleach of the vibrational bands of **1** and the simultaneous appearance of a small band at 2002 cm^–1^ and a pronounced set of peaks at 2013, 1907, and 1884 cm^–1^ (Figure 5 and Table 1). These peaks are convoluted with the bleach of **1**; however, the clean spectrum was obtained by other means discussed below. With reference to previous work, the band at 2002 cm^–1^ is tentatively assigned to the radical anion, **1**^•–^, which should have additional bands near 1890 and 1970 cm^–1^.^13,62,63^ The species with ν_CO_ = 2013, 1907, and 1884 cm^–1^ is assigned as **1**-**OPh** along the proposed ECEEC′ pathway. The complex is a neutral Re(I) species, and these data are in good agreement with the singly reduced form of [Re(pdbpy)(CO_3_)Cl], which underwent the same proposed reaction. The frequencies of this species are similar to the reference data^63^ of neutral radical solvento complexes, [Re(bpy)(CO)_3_(CH_3_CN)]^•^, but this possibility was ruled out by an acid/base reaction of **1** with (iPr)_2_NH, which cleanly produced **1-OPh** (Figure S16). Moreover, solvento radicals often exhibit two broad IR bands experimentally, and the computed IR frequencies of the corresponding neutral radical solvento complex [Re(pmbpy)(CO)_3_(CH_3_CN)]^•^ (2014, 1918, 1899 cm^–1^) do not fit. The IR spectrum of **1-OPh** in the low energy IR region matches well the DFT computed spectrum of this species (Figure 6). Vibrations around 1600 cm^–1^ can be assigned to C–C stretches of the bpy ligand. The band at 1532 cm^–1^ is mainly due to C–C stretching modes of the bpy-Ph ligand. Two peaks at 1479 and 1469 cm^–1^ are in-plane C–H bending modes of bpy-Ph, and the 1400 cm^–1^ peak is due to strongly coupled C–C and C–O stretching modes of the phenolate ligand. While overall charge and the oxidation state of the metal center in **1** and **1-OPh** are the same, observed shifts of ligand vibrational modes are consistent with significant distortion of phenyl-bpy ligand due to the binding of the phenolate to the Re metal center. This observation is also consistent with significant shifts of ^1^H NMR signals of the bpy ligand upon the formation of **1-OPh** (see below).

**Table 1:** Selected Experimental and Calculated ν_CO_ Vibrational Frequencies of **1**, Its Reduced Forms, and Related Complexes in Acetonitrile^a^

	ν_CO_/cm^–1^		
	experimental	calculated	
[Re(pmbpy)(CO)_3_Cl] (**1**)	2022, 1919, 1895	2022, 1921, 1904	*cis*
		2018, 1917, 1895	*trans*
[Re(pmbpy)(CO)_3_Cl]^•–^ (**1**^•–^)	2002	2001, 1893, 1877	*cis*
		1997, 1889, 1868	*trans*
**1-OPh**	2013, 1907, 1884	2011, 1903, 1891	
**1-OPh**^•–^	1990, 1872, 1858	1989, 1873, 1864	
**1-PhO(CH**_**3**_**CN)**^**2–**^	1995, 1873, 1858	1970, 1874, 1843	*cis*
**1-PhO**^**2–**^		1935, 1844, 1823	*cis*
[Re(bpy)(CO)_3_(imidazolate)]	2018, 1908*^a^*		
[Re(bpy)(CO)_3_(imidazolate)]^•^^–^	1995, 1878*^a^*		
**1-CH**_**3**_**CN**^**+**^	2039, 1942, 1926	2036, 1946, 1929	*cis*
**1-CH**_**3**_**CN**^**•**^	2022, 1912, 1893	2014, 1918, 1899	*cis*
**1-PhO(CH**_**3**_**CN)**^•–^	2000, 1909, 1876	2005, 1909, 1881	*cis*

a All assignments are also made in analogy with species already reported in the literature.^13,14,65^

Stepping the electrolysis potential by only −100 mV to −1.6 V immediately produced a mixture of **1**^•–^, **1**-**OPh**, and a new species with ν_CO_ = 1990, 1872, and 1858. The new species, assigned as **1-OPh**^•–^, is the sole product of electrolysis at −1.7 V, which is very close to *E*_1/2_ of the **1**^**0/–**^ couple in agreement with simulations. Here, the parallel between **1**-**OPh**, **1**-**OPh**^•–^, and the reductively deprotonated imidazolate complex and its subsequent radical in the literature is noteworthy (Table 1).^64^ The reductive chemistry was further probed by briefly electrolyzing the sample, stopping electrolysis, and continuing IR data collection. These experiments show that **1**^•–^ dissociates chloride and converts to **1**-**OPh**, without electrolysis verifying the chemical nature of this step.

According to CV data, **1**-**OPh**^•–^ is reduced at −2.26 V (peak III). IR-SEC at this potential induced small blue shifts in ν_CO_ to 1995, 1873, and 1858 cm^–1^ inconsistent with calculated values of 1960, 1839, and 1830 cm^–1^ for **1**-**OPh**^**2–**^ with retention of the chelate or of 1935, 1844, and 1826 cm^–1^ for the 5-coordinate complex **1**-**PhO**^**2–**^ with a dissociated Re–O bond. Instead, we propose that the product of peak II is the solvento complex **1**-**PhO(CH**_**3**_**CN)**^**2–**^ with a dangling phenolate group. While dissociation of the anionic ligand is expected, the affinity for solvent in this highly reduced state is unexpected and has implications regarding catalysis as will be discussed below. Computed frequencies of the final product are an imperfect match. However, it is worth mentioning that even a single CH_3_CN molecule explicitly included in the calculations has a significant effect on the computed frequencies, and an extensive survey of other possibilities did not provide promising alternatives.

Reduction of **1** to **1-OPh, 1-OPh**^•–^, and **1**-**PhO(CH**_**3**_**CN)**^**2–**^ was also investigated using sodium amalgam with UV–vis detection (Figure S17). The first reaction cleanly produced **1-OPh** as a pale-yellow solution after reaction of the phenol with sodium and substitution of chloride for phenoxide. Reduction to **1-OPh**^•–^ was indicated by intense structured bands at 500 nm and a broad band at 650 nm (the complex is dark purple). A final change indicated by a shift in isosbestic points and characterized by growth of radical absorptions was observed for the reduction to **1**-**PhO(CH**_**3**_**CN)**^**2–**^. Both **1-OPh**^•–^ and **1**-**PhO(CH**_**3**_**CN)**^**2–**^ are expected to have ligand radical character, and similar transitions are observed for both species. Identities of each step were confirmed by stopping the experiment at appropriate stages and transferring the sample to a sealed IR cell under an inert atmosphere. The ν_CO_’s were identical to those obtained during electrolysis. **1**-**PhO(CH**_**3**_**CN)**^**2–**^ is strongly reducing and slowly oxidized back to **1-OPh**^•–^. Separately, **1-OPh**^•–^ was oxidized to **1-OPh** by exposure to air.

The IR and amalgam data support the proposed “slow scan” EECEC′ pathway of Scheme 2 in which **1**^•–^ undergoes reductive deprotonation and dissociation of Cl^–^ to produce **1-OPh**. The mechanism of reductive deprotonation is unclear and is counterintuitive because a reduced complex should be more basic, not more acidic, but there are now several precedents of this reaction in the literature.^13,14,39,43,66^ A theoretical approach to understanding the mechanism in Ru and Re complexes proposed a pathway involving protonation of the bpy ligand at carbon followed by disproportionation with a net loss of 1/2 H_2_.^43,67^ In agreement with the loss of H_2_ and not H^+^, electrolysis of **1** under Ar produced ∼0.45 equiv of H_2_.

To investigate the mechanism and any role of Cl^–^ in the reductive deprotonation, CV’s and IR-SEC of the solvento complex, **1-CH**_**3**_**CN**^**+**^, were measured. The CV (Figure S20) shows a reversible **1-CH**_**3**_**CN**^**+/0**^ couple at −1.48 V, and the **1-OPh**^**0/–**^ couple expected at −1.65 V confirms formation of the chelate in the absence of chloride. The IR-SEC (Figure S21) confirmed the existence of the neutral solvent radical **1-CH**_**3**_**CN**^**•**^ and interestingly showed that this species is present at a much higher concentration than **1-Cl**^•–^ upon initial reduction, although **1-OPh** is also present immediately. This observation suggests that the rate of reductive deprotonation is attenuated by the less cathodic standard potential for the solvento complex and may support the previously proposed pathway involving ligand-based protonations. Stepping the potential resulted in the presence of a minor species with red-shifted ν_CO_ consistent with deprotonation prior to coordination, i.e., **1-PhO(CH**_**3**_**CN)**^•–^ (2000, 1909, 1876 cm^–1^), although this species was observed as a mixture, and no clear CV wave was isolated. Changes during further reduction were identical to the experiment starting with **1**.

During reduction of **1**, the immediate deprotonated species with a dangling phenolate was not observed by IR. Therefore, we cannot determine whether formation of the phenolate or chloride dissociation is rate limiting; however, it is clear that a combination of H_2_ loss and the chelate effect of **1-OPh** destabilize the singly reduced **1**^•–^, which is observed only in small concentrations by IR-SEC. The IR data also confirm that **1-OPh** is reduced at a very similar potential to **1**, which is logical considering their identities as neutral Re(I) complexes. The final reduction observed by CV(III) produces **1**-**PhO(CH**_**3**_**CN)**^**2–**^. This couple is observed at fast and slow rates confirming that the chemical step of the fast scan EECEC′ is a composite Cl^–^ dissociation, CH_3_CN binding, and reductive deprotonation.

In summary, the electrochemical mechanisms as observed by CV and characterized by IR-SEC and Na-Hg reduction occur by competing EECEC′ (waves I, II, C, III, C′; isolated at fast scans) and ECEEC′ (I, C, I′, III, C′; isolated at slow scans) pathways with the chemical steps in the slow limit being a composite of reductive deprotonation, chloride dissociation, and oxygen coordination to the metal center. Importantly, the I/IV couple of **1**^**0/–**^ includes the **1-OPh**^**0/–**^ couple at slow scan rates and after a full three-electron sweep, peak IV is the reoxidation of the **1-OPh**^•–^, not **1**^•–^.

### Isolation of Complex **1-OPh**

As discussed in the Introduction, one hypothesis of this work was that a simplified system with a single pendent −OH group could confirm the validity of the mechanism shown in Scheme 1 by obtaining additional characterization of the species assigned as **1-OPh**. The IR-SEC data, supported by DFT calculations, suggest the presence of this intermediate after reduction of **1** similarly to the reduction of [Re(pdbpy)(CO)_3_Cl]. As a neutral Re(I) complex, **1-OPh** should be sufficiently stable for preparation by electrolysis and analysis by NMR.

**Figure 7:**
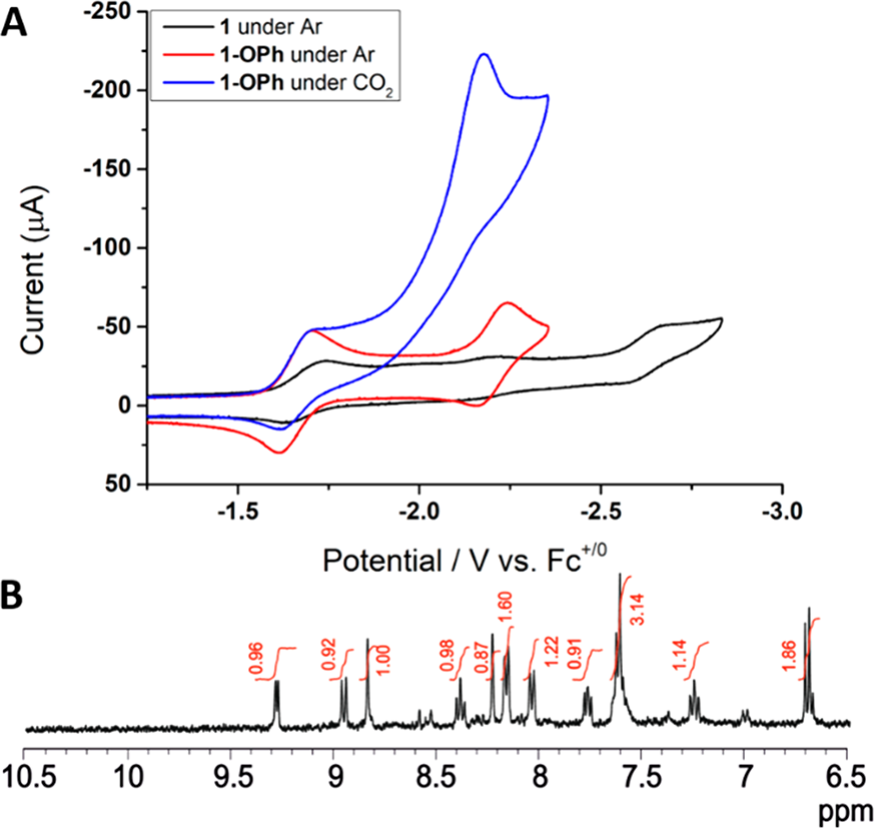
(A) CVs of **1** in acetonitrile/Bu_4_NPF_6_ before and after controlled potential electrolysis (CPE at −1.8 V vs Fc^+/0^) at 100 mV s^–1^. The working electrode was a 5 mm glassy carbon electrode. (B) ^1^H NMR spectra of **1-OPh** in *d*_6_-DMSO.

In order to accomplish this goal, we took advantage of the clean formation of **1-OPh** as ascertained by IR-SEC and increased the reaction volume to the preparative scale. Upon bulk electrolysis in CH_3_CN at the first reduction potential in a N_2_-filled glovebox, the solution changed color from yellow to dark green and the flow of current ceased. It is known from the amalgam experiment that the dark green solution is an indication of slight over reduction. Once the electrolysis was completed, an IR spectrum showed the majority presence of **1-OPh** with a small amount of **1-OPh**^•–^ (Figure S18). The CV recorded after bulk electrolysis also confirmed completion of the reaction (Figure 7A). The second reduction peak, assigned as the irreversible reduction of [Re(pmbpy)(CO)_3_Cl]^−^ is absent, and the **1-OPh**^**0/–**^ couple is observed to be nearly identical to the **1**^**0/–**^couple. The solvent was removed under reduced pressure to afford a solid, which was poorly soluble in CH_3_CN but sufficiently soluble in *d*_6_-DMSO for NMR characterization.

The ^1^H NMR spectrum of the resultant product **1-OPh** (Figure 7) reveals the absence of the −O*H* resonances and distinct changes in the chemical shifts of the protons of the phenolate ring (see SI for additional data, Figures S5–S8). Chemical shifts of the protons of the bipyridine rings also shift significantly, presumably due to the strained puckered conformation of the tridentate chelate relative to the planar orientation in **1**. The carbonyl region of the ^13^C spectrum (Figure S7) shows three unique CO resonances relative to those of **1**. A new and distinct resonance is observed at 172 ppm in a frequency region where the ^13^C spectrum of **1** has no signals. This new resonance is tentatively assigned to the quaternary carbon atom connected to the phenolate oxygen, which now coordinates the rhenium metal. Its appearance at 172 ppm is consistent with *C*=O character of the phenolate resonance structure. Another significant difference from compound **1** is the absence of signals below 118.50 ppm due to the different chemical environment experienced by CH groups in the phenolate ring of **1-OPh**.

### Reactivity toward CO_2_

**Figure 8:**
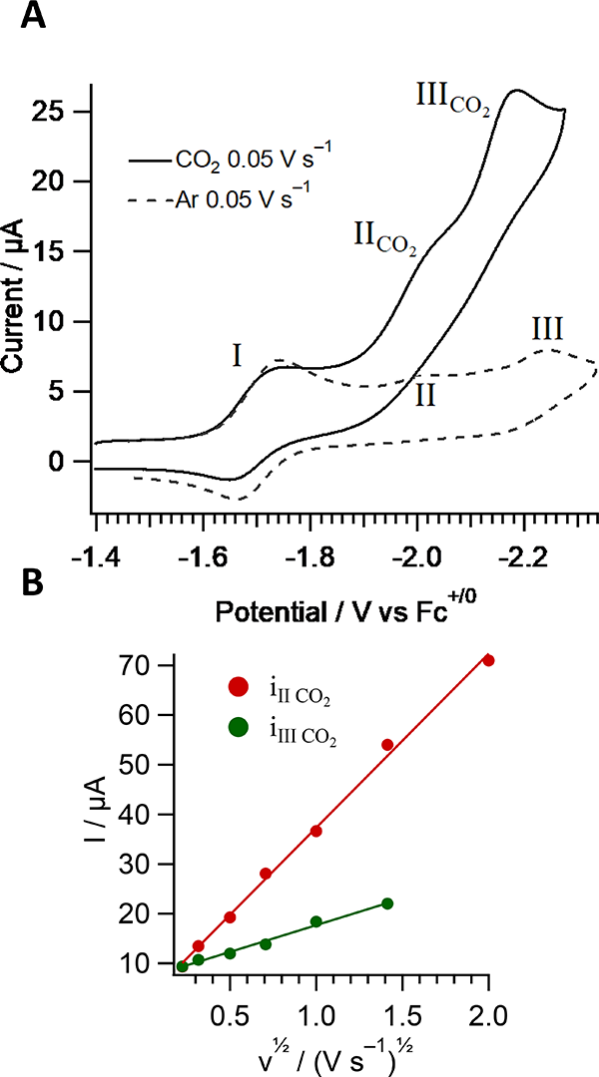
(A) CVs of 0.4 mM **1** in Ar or CO_2_-saturated CH_3_CN with 0.1 M Bu_4_NPF_6_ at a glassy carbon electrode, scan rate = 50 mV s^–1^. (B) Current at peaks II_CO_2__ and III_CO_2__.

Complex **1** was evaluated as a homogeneous electrocatalyst for CO_2_ reduction in CH_3_CN by CV and bulk electrolysis. In the presence of CO_2_, the CV shows a catalytic current beginning at wave II and an increase in slope at wave III (Figure 8). The solution of **1-OPh** prepared *in situ* shows similar behavior in Figure 7. The nature of this current enhancement was investigated at various scan rates. Eq 1 is derived for the condition of kinetic control with no substrate consumption (i.e., the rate of consumption is countered by the rate of diffusion), where the catalytic current is “S-shaped” and *i*_c_ is read as the plateau current.^68^ According to eq 1, where *n* = 2, *F* is Faraday’s constant, *A* is the electrode area, *D* is the diffusion coefficient, *k* is the rate constant, and *a* is the reaction order with respect to CO_2_; an electrocatalytic current should be scan-rate independent provided the necessary conditions are met.
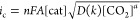
1

Data in Figure 8, panel B, show that the additional current observed under CO_2_ is proportional to ν^1/2^ over the entire range of data collection, suggesting that the electrochemical currents here are controlled by electron transfers (eq 2), and that catalysis is slow on the CV time scale. For comparison, the same experiment was performed on the reference compound, [(4,6-diphenyl-2,2′-bipyridine)Re(CO)_3_Cl], which shows a catalytic current that becomes scan rate-independent above 2 V s^–1^ (Figure S22). The total current at II_CO_2__ and III_CO_2__ relative to peak I is a maximum of 4 at slow scan rates but converges to 3 as the sweep rate exceeds 250 mV s^–1^, suggesting that CO_2_ may bind to the metal in a reduced state, but a competing reaction is limiting the turnover frequency.
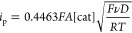
2

Bulk electrolysis in CO_2_-saturated CH_3_CN was performed to examine the products of catalysis. The potential was cathodic of peak II or at peak III, and experiments were performed in dry solvent or with 5% Brønsted acids (water, methanol and phenol). Table 2 summarizes results obtained by these CPE experiments. Fewer than three turnovers were obtained in dry solvent or in the presence of water or methanol. An experiment in the presence of phenol performed slightly better, yet turnovers were underwhelming. Electrolysis at wave III (−2.3 V vs Fc^+/0^) yielded nine turnovers; however, the catalyst ceased activity faster at this strongly reducing potential.

**Table 2:** Bulk Electrolysis Data of 0.5 mM Solution of **1** in Acetonitrile

*E*/V^a^	time/min^b^	acid^c^	TON_CO_	FE_CO_%
–2.0	75		1.4	100
–2.0	160	H_2_O	3	98
–2.0	60	CH_3_OH	1.2	100
–2.0	150	phenol	4	104
–2.3	100	phenol	9	102

a V vs Fc^+/0^.

b Time before activity ceased.

c 5% by volume.

**Scheme 3:**
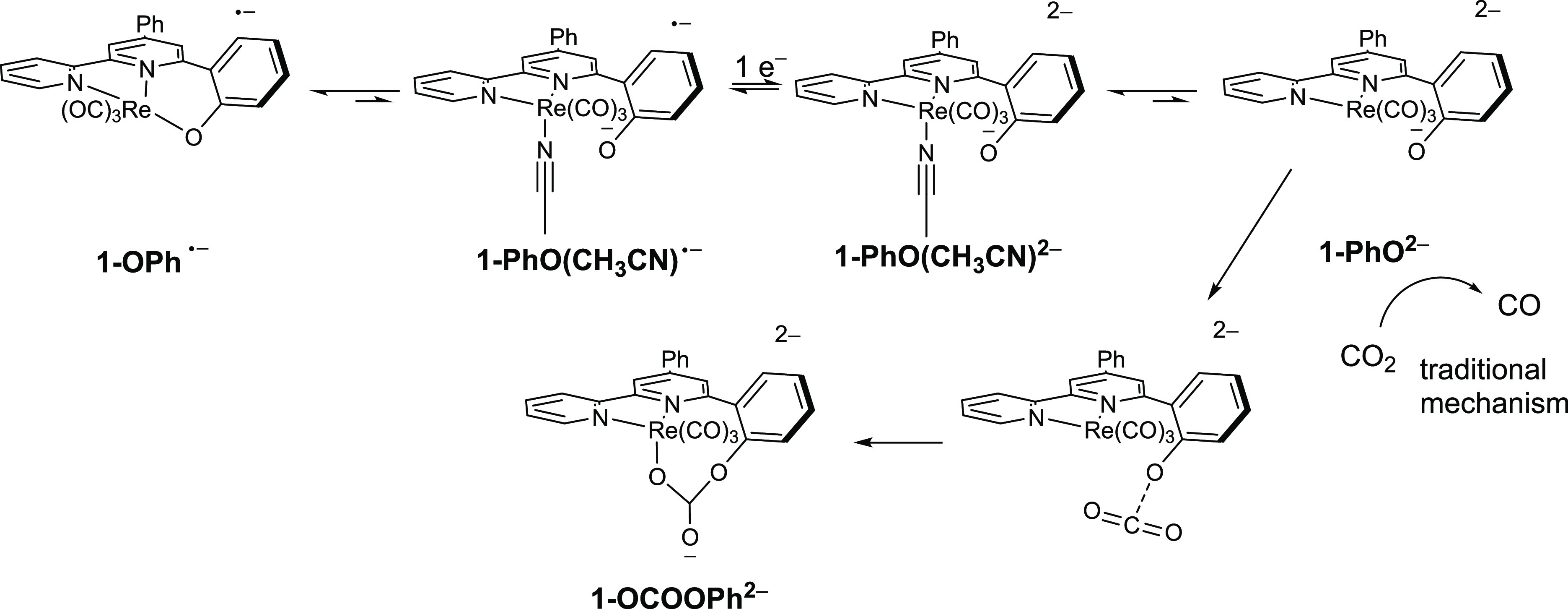
Proposed Electrochemical Mechanisms for Reduction of **1** in the Presence of CO_2_

The catalytic reactivity is interpreted according to Scheme 3. When **1-OPh** is reduced to **1-OPh**^•–^, a small amount of the solvento complex, **1-PhO(CH**_**3**_**CN)**^•–^, may exist in equilibrium. At the potential of the **1-OPh**^**0/–**^ couple, the solvento complex will be reduced immediately to the dianion, **1-PhO(CH**_**3**_**CN)**^**2–**^. Evidence for this reaction was found during chemical reduction in which samples partially reduced from **1-OPh** to **1-OPh**^•–^ showed small shoulders assignable to **1-PhO(CH**_**3**_**CN)**^**2–**^. This equilibrium is uphill by 51.7 kJ mol^–1^ and inhibits catalysis at peak II. Direct reduction of **1-OPh**^•–^ at peak III produces **1-PhO(CH**_**3**_**CN)**^**2–**^, which explains the increased catalytic current at peak III; however, overall catalysis is inefficient for two reasons: (1) the strong affinity of the dianion for solvent and (2) reactivity of CO_2_ at the oxyanion. The latter is predicted by the Mulliken and Hirshfeld atomic charges of **1-PhO**^**2–**^, which predict that the phenolate is the most nucleophilic site. The reaction with CO_2_ is mildly endergonic, but the rearrangement to the chelating carbonate **1-OCOOPh**^**2–**^ is exergonic by 21.2 kJ mol^–1^. Identification of the precise pathway for CO production is computationally uncertain; however, we propose that CO_2_ which interacts with the Re center of **1-PhO**^**2–**^ may convert to CO via standard pathways.^29^ The **1-OCOOPh**^**2–**^ may release CO; however, this species is likely stable and may reduce catalytic rates. The “slow one-electron pathway”^29^ is also characterized by an intermolecular Re–O bond (which is stronger than Re–C bond), but **1-OCOOPh**^**2–**^ displays an entropically stabilized intramolecular Re–O bond. The IR-SEC of **1** under a CO_2_ atmosphere produces spectra very similar to those observed under N_2_, indicating that the steady state concentration of any CO_2_ adduct intermediate is below the detection limit of the experiment.

Catalytic performances of **Re-2OH** with respect to **1** are only marginally better, yielding a TON_CO_ of 14, whereas the presence of three OH groups in meta and para phenolic positions decrease the catalytic activity toward CO_2_ reduction,^13^ thus confirming that despite the close proximity of the OH group to the metal center in which chelation of a carbonate can be inhibitive, the close metal–OH proximity also has beneficial effects. Similar proximity conclusions were drawn by Bocarsly on **Mn-1OH**, which showed enhanced reactivity attributed to better hydrogen bonding compared to related congeners with distal OH groups.^23^ Mn(bpy)(CO)_3_Br reported by Deronzier et al.^17^ displayed a TON_CO_ of 13, while **Mn-1OH** showed a reduced TON_CO_ value of 2.7.^45^ Conversely, our **Mn-2OH**^14^ yielded a higher TON_CO_ (28) together with formate production (TON_HCOO_^–^ 12). Interestingly their predicted catalytic mechanism involves the formation of a hydrogen bond between the phenolic proton and the Mn-bound CO_2_ molecule, followed by facile proton-assisted C–O bond cleavage of the CO_2_ to form CO.^23,45^ Contrarily, our investigations on **Mn-2OH** show that the reductive deprotonation of phenolic OH takes place before CO_2_ binding to the Mn center, similar to complex **1** in the present study. Meanwhile, the effect of a second coordination sphere^69^ has been investigated for a Mn complex, {Mn^I^([(MeO)_2_Ph]_2_bpy)(CO)_3_(CH_3_CN)}(OTf), reported by Rochford et al.,^70^ where [(MeO)_2_Ph]_2_bpy = 6,6′-bis(2,6-dimethoxyphenyl)-2,2′-bipyridine. Together with the steric influence preventing dimerization,^71^ the four pendent methoxy groups exhibit weak hydrogen bonding interaction, playing a significant role during CO_2_ reduction. Complexes **1**, **Re-2OH**, and **Mn-2OH** prevent the dimerization too, by forming the intramolecular chelating M–O (M = Re, Mn) bond, but the resulting Re–O bond appears to be strong enough to limit the catalytic activity toward CO_2_ reduction, or formation of a carbonate may also limit catalysis. On the other hand, Mn-based catalysts provide better TON values than their precious metal congeners, probably because greater strain in the Mn–O bond leads to less stability of the intermediate.

## Conclusions

This study provides an intimate understanding of the electrochemical behavior of *fac*-Re(pmbpy)(CO)_3_Cl (**1**). NMR data suggest the existence of two different isomers, **1-***cis* characterized by the proximity of Cl and OH moieties and **1-***trans*, where Cl and OH are oriented *trans* to each other. DFT calculations confirmed that a non-negligible energy barrier separates the two isomers and reveal two transition states, ^**TS**^**1′** and ^**TS**^**1′′**, with activation energy of 75.5 and 95.0 kJ mol^–1^, respectively. By experimental evidence, we confirmed the tendency toward the formation of **1-OPh** bearing a Re–O intramolecular bond. **1-OPh** has been synthesized by controlled potential electrolysis and characterized by ^1^H NMR spectroscopy, which revealed the absence of the OH resonances and distinct changes in the chemical shifts of the protons of the phenolate and bpy rings when compared to **1** due to coordination of the phenolate and a puckered distortion of the bpy backbone. The IR spectrum of isolated **1-OPh** exhibits three new CO stretching bands (2013, 1907, 1884 cm^–1^) which exactly overlap with the bands obtained after the first 1e^–^ reduction during IR-SEC, in agreement with DFT calculations. The CV recorded after bulk electrolysis added further evidence of the formation of **1-OPh** and confirmed competition between EECEC′ and ECEEC′ pathways for the reduction of **1**. After electrolysis, the peak assigned to the irreversible reduction of **1**^•–^ is absent, and the **1-OPh**^**0/–**^ couple is observed at a potential nearly identical to the **1**^**0/–**^ redox couple. Similar intermediates, as depicted in Scheme 1, were already proposed to be formed during electrochemical reductions of the closely related **Re-2OH** and **Mn-2OH**. The electrocatalytic properties of these two complexes were deeply investigated in our previous works. As we previously concluded, the effect of the local proton source is more beneficial in terms of electrocatalytic turnovers by Mn compared to Re and also changes the selectivity for CO and formate. Previous conclusions are consistent with the low CO turnover numbers of **1** and the absence of formate as a reduction product. We can conclude that a reductive deprotonation of **1** affords **1-OPh**, evidencing the capability of the pendent OH group to behave as a chelating ligand. Two subsequent reductions are needed to provide the active catalyst; however, a small equilibrium dissociation of the Re–O bond after the first reduction provides a minor pathway for catalysis at a lower overpotential. The active catalyst is a dianion with a dangling phenolate that strongly favors solvent coordination over a five-coordinate intermediate. This equilibrium disfavors catalysis, and calculations suggest that the oxyanion is more nucleophilic toward CO_2_ than the Re(0) center. Formation of a stable carbonate and metal chelation lead to diminished CO_2_ interaction with the reduced complexes consistent with small CV catalytic currents and a low TON_CO_ under CO_2_. The mechanistic characterization and comparison to related complexes highlight the importance of considering deleterious reactivity by coordination or ligand-based reactivity of CO_2_ when designing catalysts with pendent acid/base functionality in the second coordination sphere; however, the greater reactivity of the same ligand set on Mn is a promising observation considering that the (L)M(CO)_3_X motif is one of a few examples where first and third row metal congeners with the same ligand set undergo similar CO_2_ conversion reactions.

## Supporting Information Available

Data include NMR spectra, cyclic voltammograms, UV–vis absorption and IR spectra, sodium amalgam reduction and DFT calculations (PDF)
