# Supplementary material for: Molecular Catalysts with Intramolecular Re–O Bond for Electrochemical Reduction of Carbon Dioxide
Source: Inorg Chem. 2020 Aug 17;59(17):12187–99. doi: 10.1021/acs.inorgchem.0c01181 (PMC8009525; doi:10.1021/acs.inorgchem.0c01181)
Supplement: Supplementary file 1 — ic0c01181_si_001.pdf [file ic0c01181_si_001.pdf]

Supporting Information

Molecular Catalysts with Intramolecular Re-O Bond for Electrochemical Reduction of Carbon Dioxide

Laura Rotundo,† Dmitry Polyansky,‡ Roberto Gobetto,† David Grills,‡ Etsuko Fujita‡, Carlo Nervi,*† Gerald Manbeck*‡

Chemistry Department, University of Torino, Via P. Giuria 7, 10125, Torino, Italy, and CIRCC (Bari)

Chemistry Division, Brookhaven National Laboratory, Upton, NY 11973-5000, USA

**contact email:* carlo.nervi@unito.it, gmanbeck@bnl.gov

Contents

I. COSY, DEPT-135 and ^{13}C NMR spectra S2

II. Cyclic voltammetry S7

III. Acid/base reaction of [Re(pmbpy)$(CO)_3$Cl] S11

IV. Sodium amalgam reduction S12

V. IR spectroscopy after preparative scale electrolysis S13

VI. *fac*-[Re(pmbpy)$(CO)_3(CH_3CN)$](PF_6) S14

VII. Cylic voltammetry of [(4,6-diphenyl-2,2′-bipyridine)Re$(CO)_3$Cl under CO_2 S16

VIII. DFT Calculations S17

I. NMR Characterization

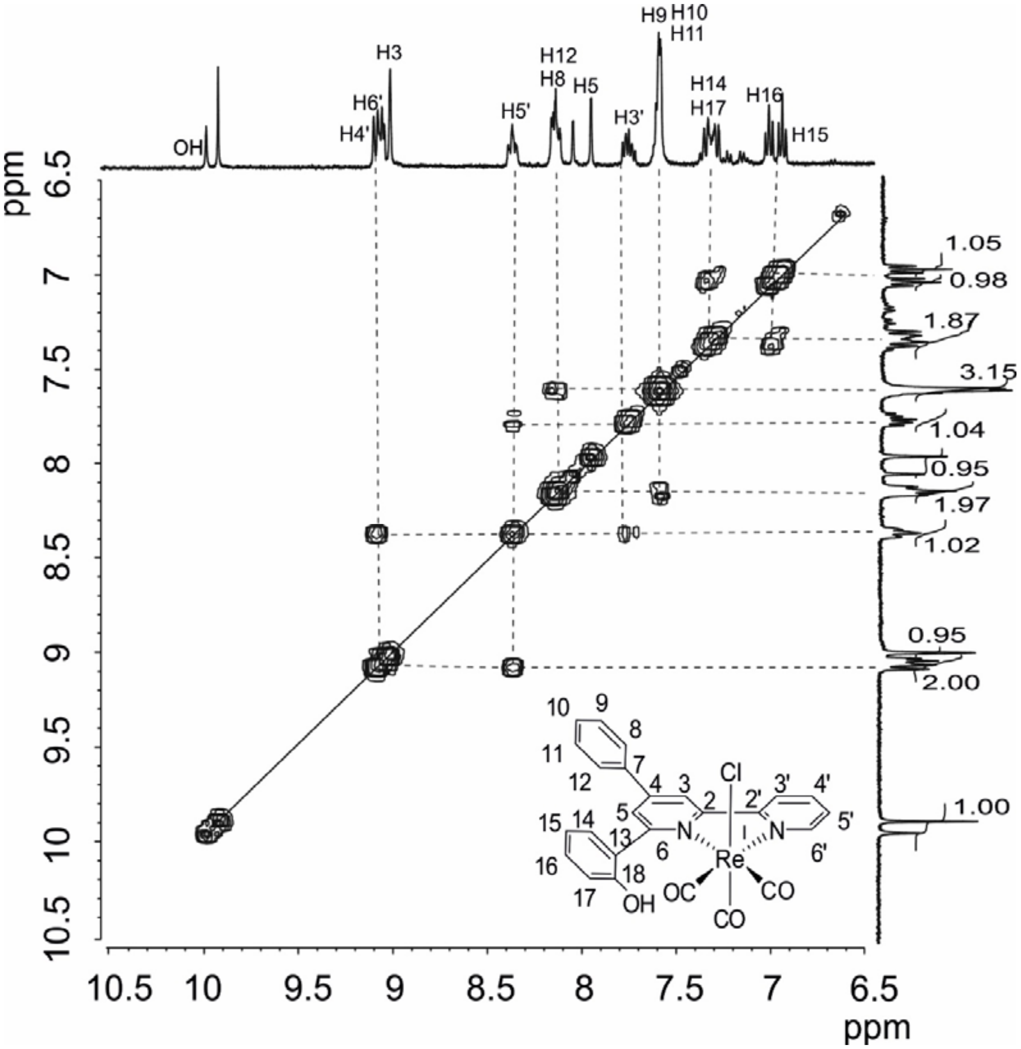

Figure S1. 2d-COSY spectrum of [Re(pmbpy)(CO)$_3$Cl] (**1**) in d_6-DMSO. Numeration in the chemical structure reported in the figure is referred to the carbon skeleton for 2,2′ bipyridines. Each –C*H* proton has been named with the respective number of the carbon.

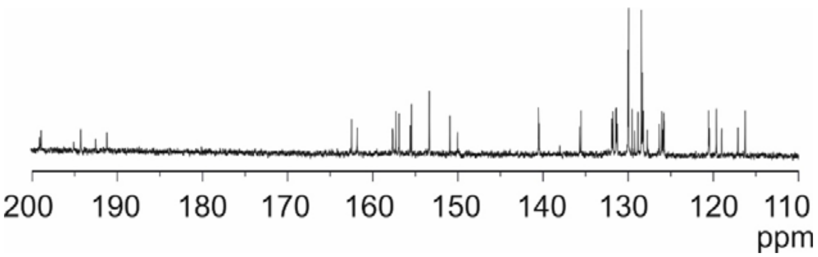

Figure S2. ^{13}C NMR spectrum of [Re(pmbpy)(CO)$_3$Cl] (**1**) in d_6-DMSO.

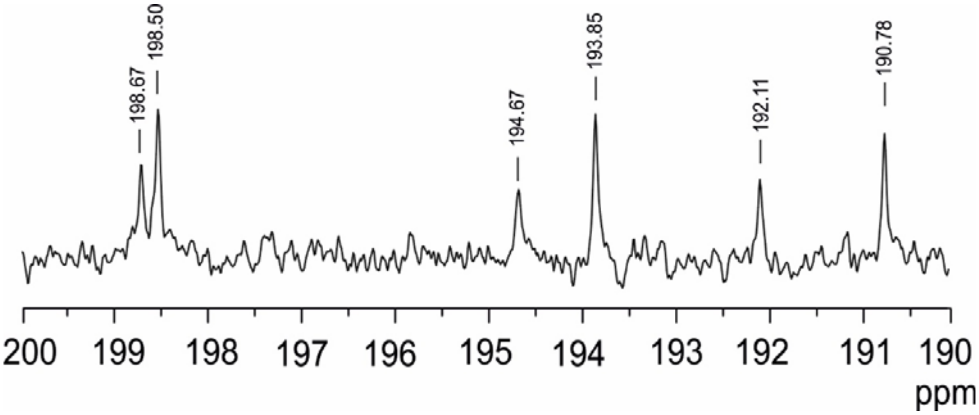

Figure S3. Expansion of the carbonyl region of ^{13}C NMR spectrum of [Re(pmbpy)(CO)$_3$Cl] (**1**) in d_6-DMSO.

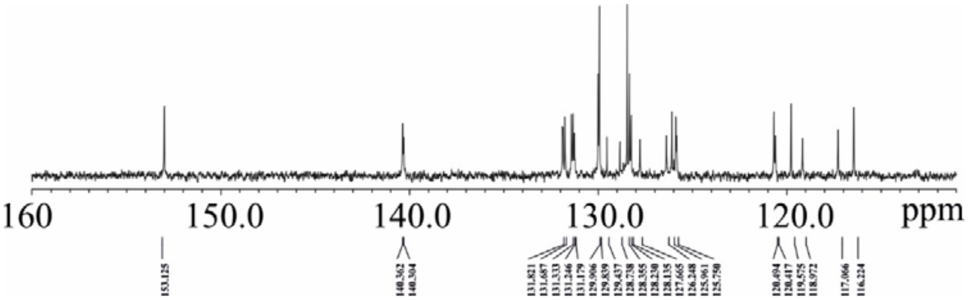

Figure S4. DEPT-135 spectrum of [Re(pmbpy)(CO)$_3$Cl] (**1**) in d_6-DMSO.

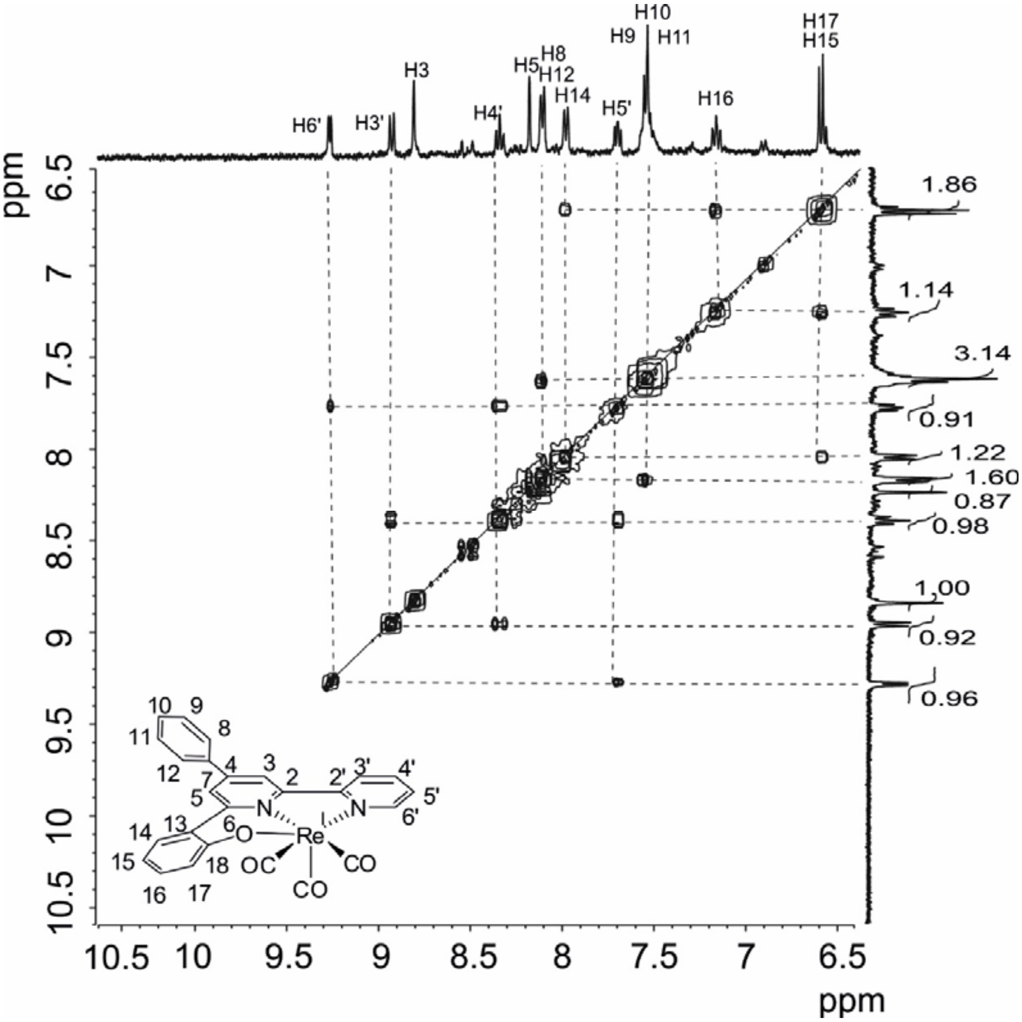

Figure S5. 2d-COSY spectrum of **1-OPh** in d_6-DMSO. Numeration in the chemical structure reported in the figure is referred to the carbon skeleton for 2,2′ bipyridines. Each –C*H* proton has been named with the respective number of the carbon.

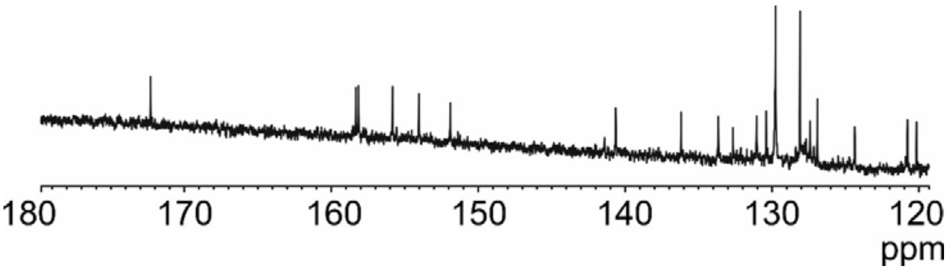

Figure S6. ^{13}C NMR spectrum of **1-OPh** in d_6-DMSO.

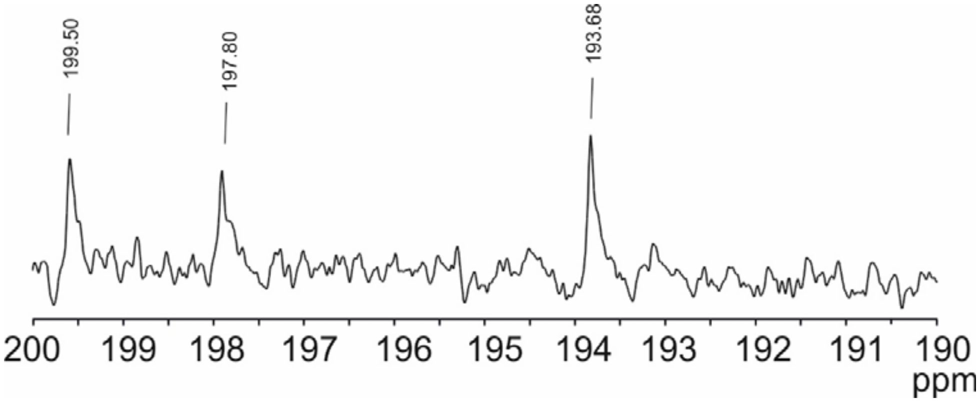

Figure S7. Expansion of the carbonyl region of ^{13}C NMR spectrum of **1-OPh** in d_6-DMSO.

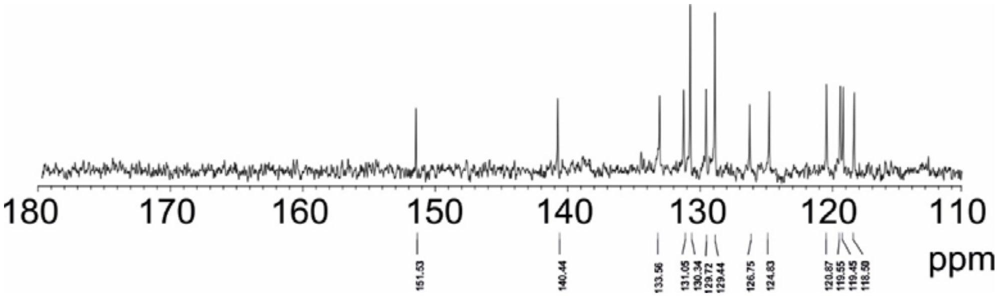

Figure S8. DEPT-135 spectrum of **1-OPh** in d_6-DMSO.

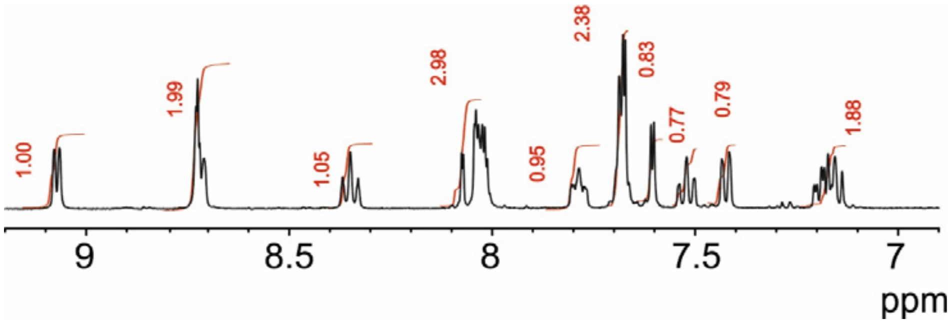

Figure S9. ^{1}H NMR spectrum of Re(pmbpy)$(CO)_3(CH_3CN)](PF_6)$ in CD_3CN.

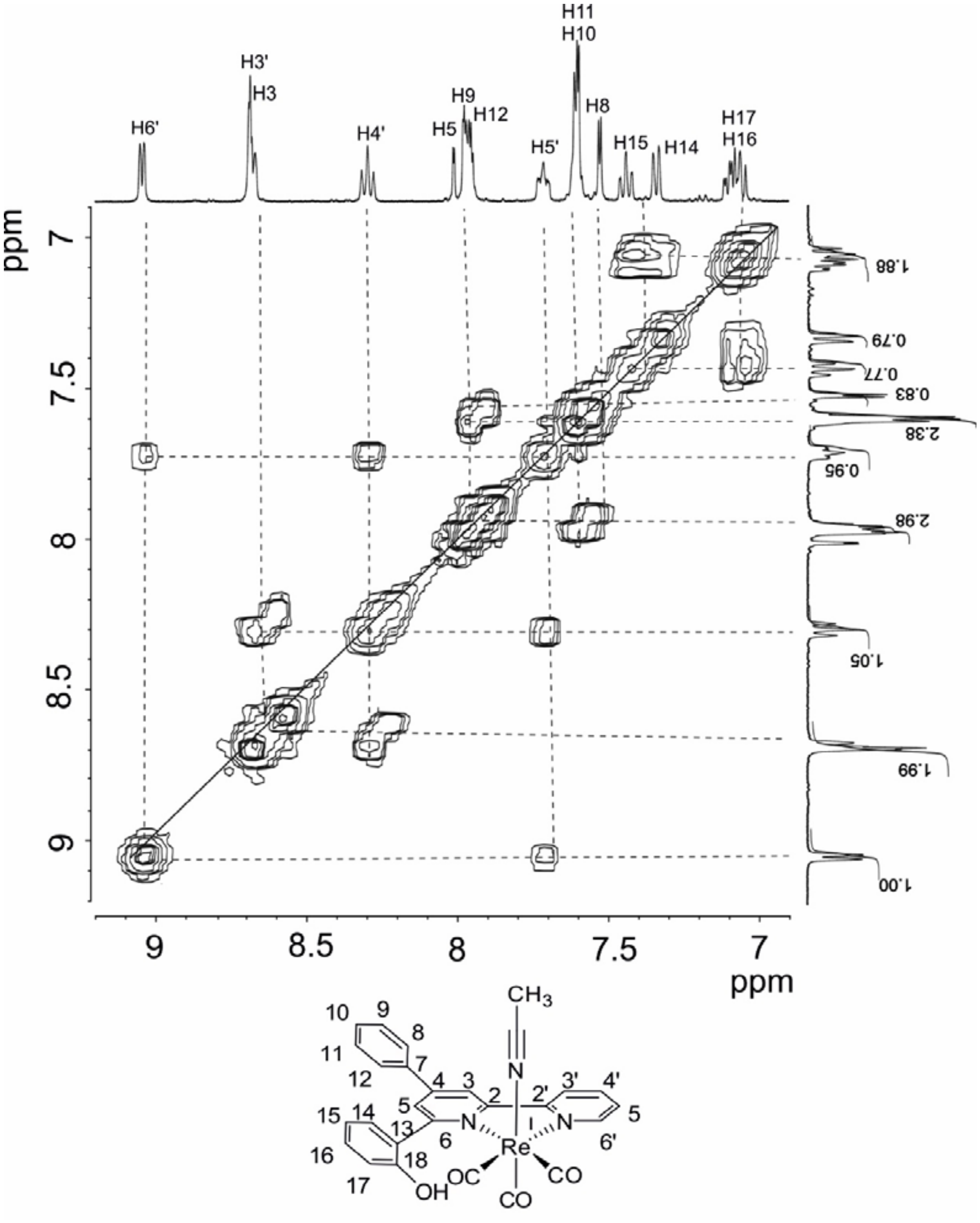

Figure S10. 2d- COSY spectrum of Re(pmbpy)(CO)$_3$(CH$_3$CN)](PF$_6$) in CD$_3$CN.

II. Cyclic voltammetry

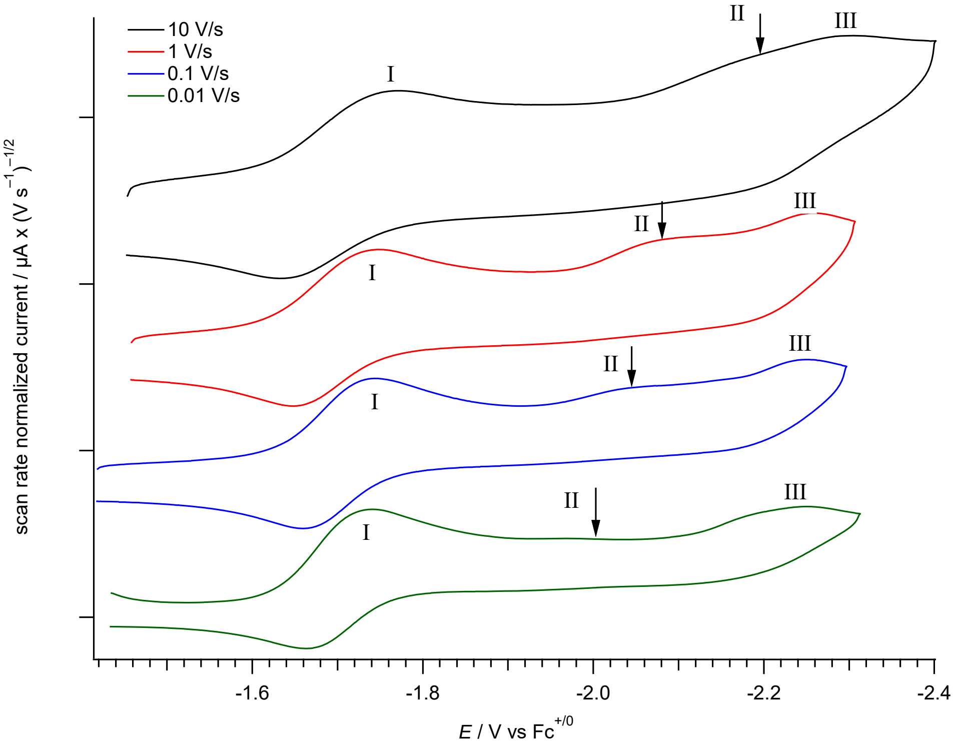

Figure S11. Current normalized cyclic voltammograms of 0.4 mM **1** in Ar-saturated CH_3CN with 0.1 M Bu_4NPF_6 at a glassy carbon electrode with varied scan rates.

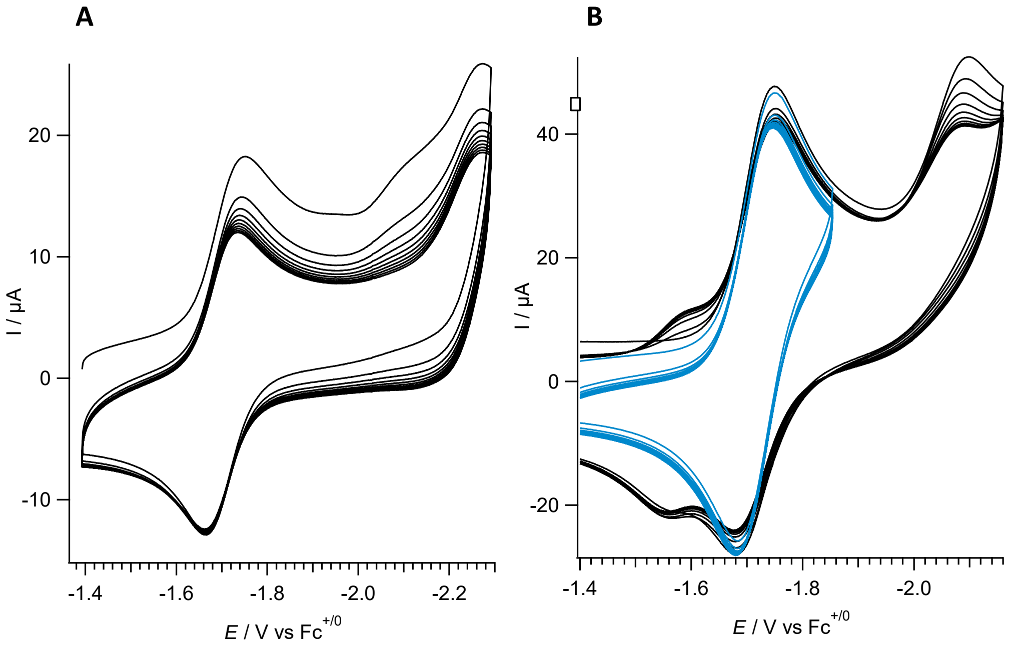

Figure S12. (A) Multisweep cyclic voltammograms of 0.4 mM **1** (A) and 1 mM [Re(4,6-diphenyl-2,2′-bpy)$(CO)_3$Cl] (B) in Ar-saturated CH_3CN with 0.1 M Bu_4NPF_6 at a glassy carbon electrode at 500 mV s^{-1}.

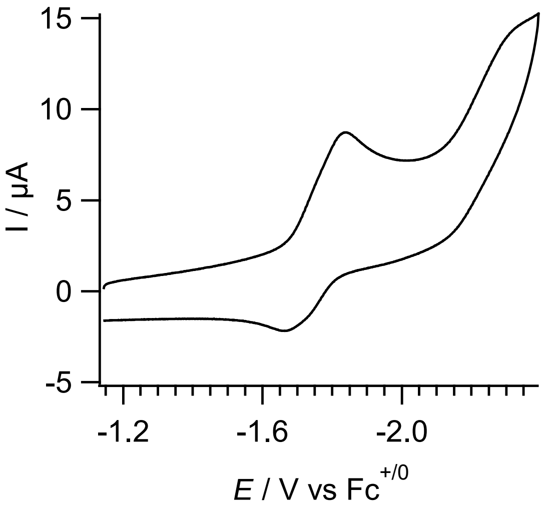

Figure S13. CV of 0.4 mM **1** in Ar-saturated butyronitrile with 0.1 M Bu_4NPF_6 at 195 K. Scan rate = 500 mV s^{-1}.

A

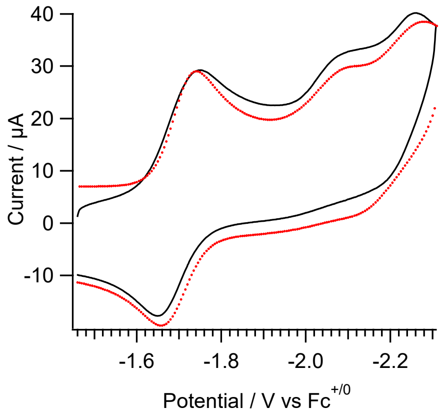

B

E $ReCl + e = ReCl^{-}$ (1)
E $ReCl^{-} + e = ReCl^{2-}$ (2)
C $ReCl^{2-} = Re\text{-}OPh^{-} + Cl^{-} + H^{\bullet}$ (3)

E $ReCl + e = ReCl^{-}$ (1)
C $ReCl^{-} = Re\text{-}OPh + Cl^{-} + H^{\bullet}$ (4)
E $Re\text{-}OPh + e = Re\text{-}OPh^{-}$ (5)

E $Re\text{-}OPh^{-} + e = Re\text{-}OPh^{2-}$ (6)
$Re\text{-}OPh^{2-} = RePhO(CH_3CN)^{2-}$ (7)

C

**Heterogeneous Reactions**	**E_o (V)**	**α (eV)**	**k_s (cm/s)**
$ReCl + e = ReCl^{-}$ (1)	−1.70	0.67	0.56
$ReCl^{-} + e = ReCl^{2-}$ (2)	−1.99	0.5	0.003
$Re\text{-}OPh + e = Re\text{-}OPh^{-}$ (5)	−1.73	0.4	0.02
E $Re\text{-}OPh^{-} + e = Re\text{-}OPh^{2-}$ (6)	−2.18	0.4	0.01
**Homogeneous Reactions**	**K_{eq}**	**k_f**	**k_b**
$ReCl^{2-} = Re\text{-}OPh^{-} + Cl^{-} + H^{\bullet}$ (3)	5.7×10^{7}	920	1.61×10^{-5}
$ReCl^{-} = Re\text{-}OPh + Cl^{-} + H^{\bullet}$ (4)	2794	0.43	1.54×10^{-4}
$Re\text{-}OPh^{2-} = RePhO(CH_3CN)^{2-}$ (7)	17	12	0.71

**Species Parameters**	**D (cm^2/s)**	**C (mM)**
ReCl	1×10^{-5}	0.39
Re-OPh	8×10^{-6}	
$ReCl^{-}$	9×10^{-6}	
$ReCl^{2-}$	2×10^{-5}	
$Re\text{-}OPh^{-}$	5×10^{-5}	
$Re\text{-}OPh^{2-}$	1×10^{-5}	
$Re\text{-}OPh^{2-}$ product	3×10^{-5}	

Figure S14. (A) Experimental CV (black) and simulated CV (red) of 0.39 mM **1** at 1 V s^{-1} in Ar-saturated CH_3CN. (B) Mechanism from Scheme 2 in the manuscript. (C) Simulation parameters.

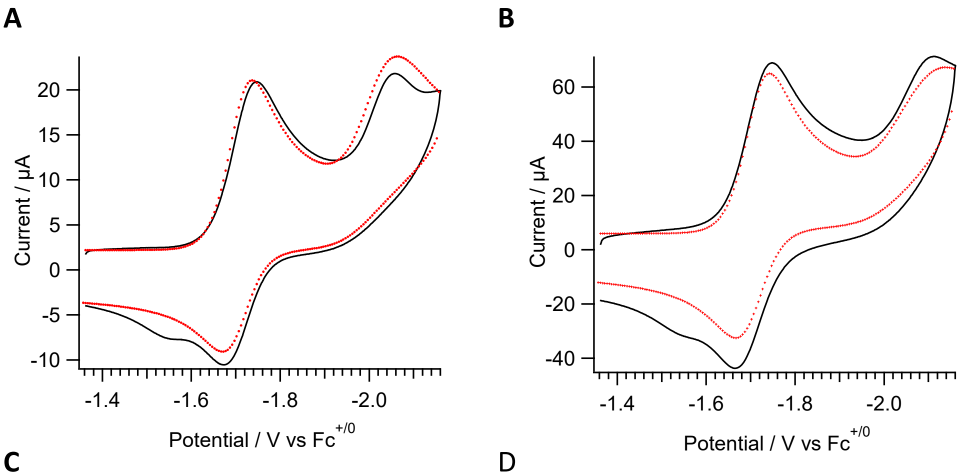

C

$ReCl + e = ReCl^-$
$ReCl^- + e = ReCl^{2-}$
$ReCl^{2-} = Re^- + Cl^-$

D

**Heterogeneous Reactions**	**E_o (V)**	**α (eV)**	**k_s (cm/s)**
$ReCl + e = ReCl^-$	−1.70	0.66	0.057
$ReCl^- + e = ReCl^{2-}$	−2.08	0.35	0.012
**Homogeneous Reactions**	**K_{eq}**	**k_f**	**k_b**
$ReCl^{2-} = Re^- + Cl^-$	5.7×10^7	900	1.58×10^{-5}
**Species Parameters**	**D (cm²/s)**	**C (mM)**	
ReCl	1×10^{-5}	1.0	
$ReCl^-$	1.4×10^{-5}		
$ReCl^{2-}$	2×10^{-5}		
Re^-	2×10^{-5}		

Figure S15. (A) Experimental CV (black) and simulated CV (red) according to an EEC reductive mechanism of 1 mM [Re(4,6-diphenyl-2,2′-bpy)(CO)3Cl] in Ar-saturated CH_3CN at 100 mV s^{-1} and (B) 1000 mV s^{-1}. (C) Mechanism. (D) Simulation parameters.

III. Acid/base reaction of [Re(pmbpy)(CO)$_3$Cl]

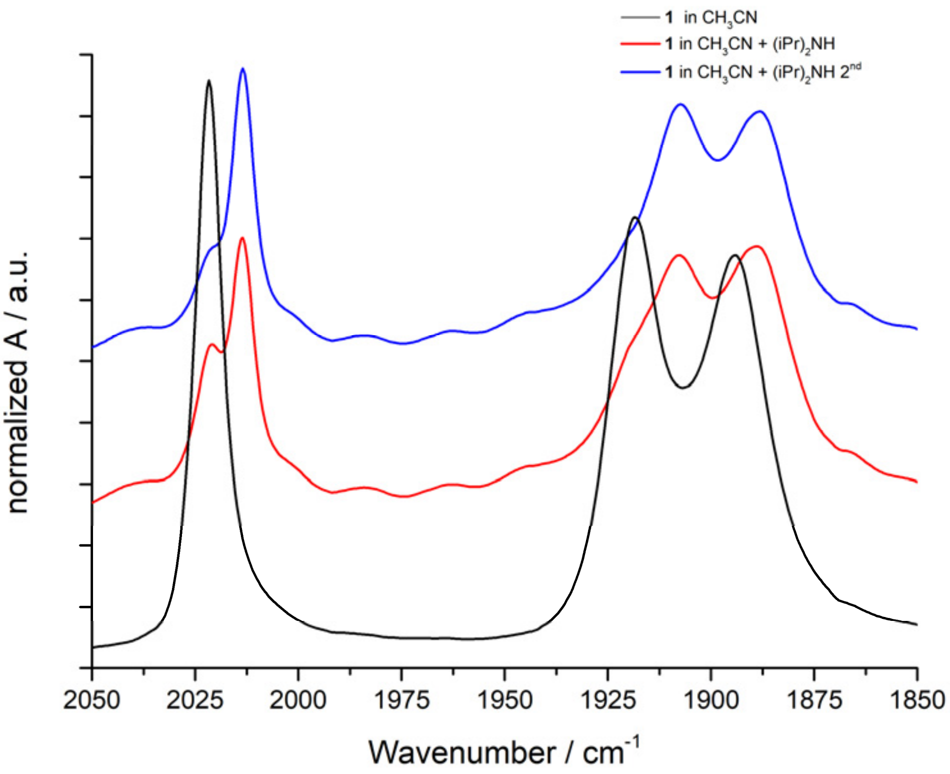

Figure S16. IR spectra before (black) and after (red and blue) addition of 0.5 mM $(iPr)_2NH$ to a 0.5 mM solution of **1** in CH_3CN. Red spectrum: equimolar addition of base; blue spectrum: excess of base was added.

IV. Sodium amalgam reduction

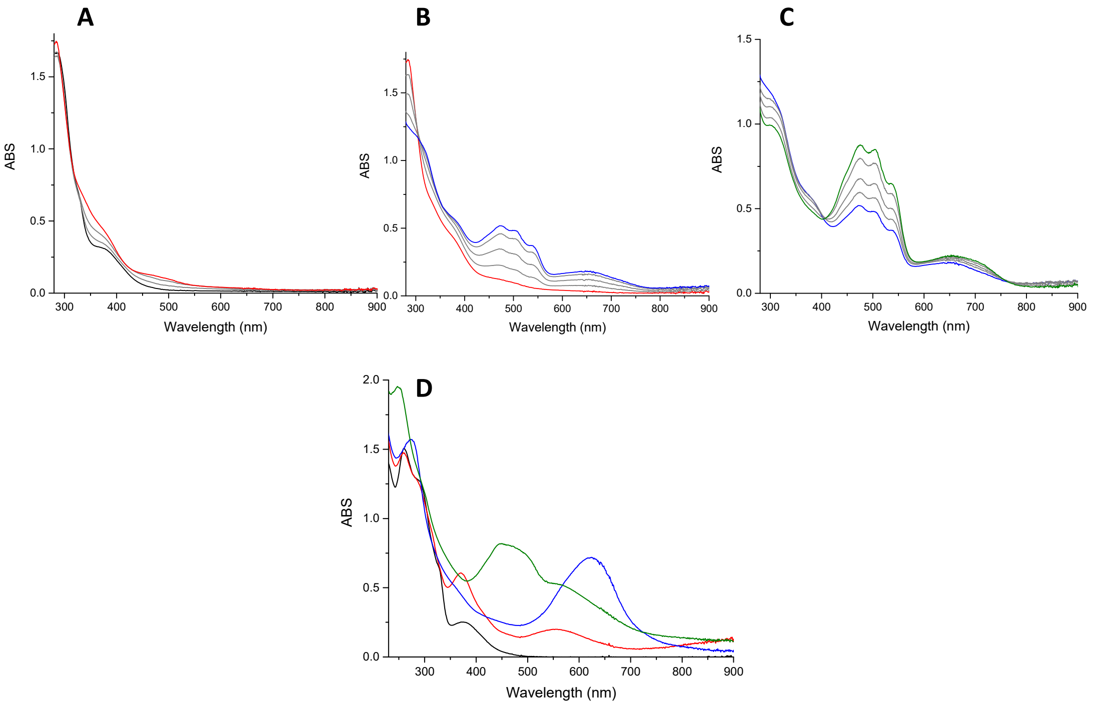

Figure S17. Sodium amalgam reduction of [Re(pmbpy)$(CO)_3Cl$] (A-C) and [Re(4,6-diphenyl-2,2′-bpy)$(CO)_3Cl$] (D). The stepwise reduction of [Re(pmbpy)$(CO)_3Cl$] produces **1-OPh** (A, red), **1-OPh**$^{\bullet-}$ (B, blue), and **1-PhO(CH_3CN)**$^{2-}$ (C, green). The species is a ligand radical with a dissociated phenolate. In (D), the starting complex is sequentially reduced to [Re(4,6-diphenyl-2,2′-bpy)$(CO)_3Cl$]$^-$ (red), [Re(4,6-diphenyl-2,2′-bpy)$(CO)_3$]$^-$ (blue), and [Re(4,6-diphenyl-2,2′-bpy)$(CO)_3$]$^{2-}$ (green).

V. IR spectroscopy after preparative scale electrolysis

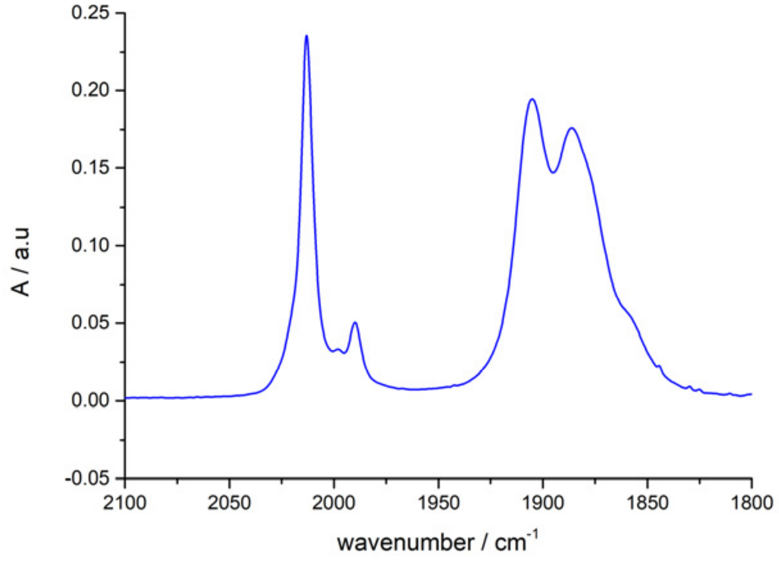

Figure S18. IR-spectrum in acetonitrile of isolated **1-OPh** species after bulk electrolysis experiment.

VI. *fac*-[Re(pmbpy)$(CO)_3(CH_3CN)](PF_6)$.

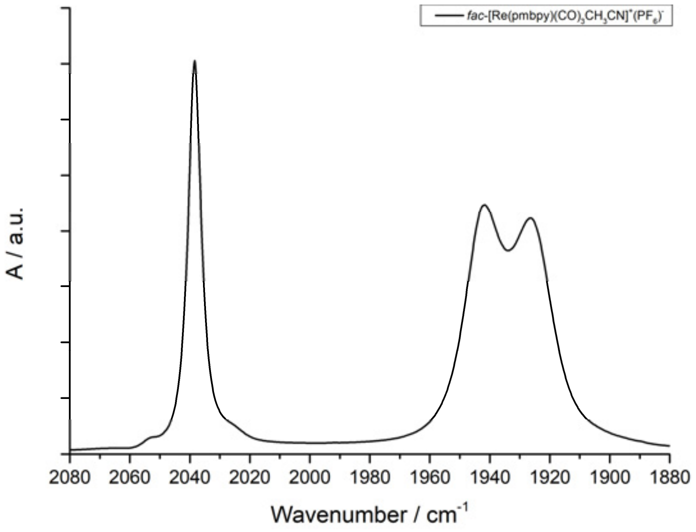

Figure S19. IR spectrum of **Re(pmbpy)$(CO)_3(CH_3CN)](PF_6)$** in CH_3CN.

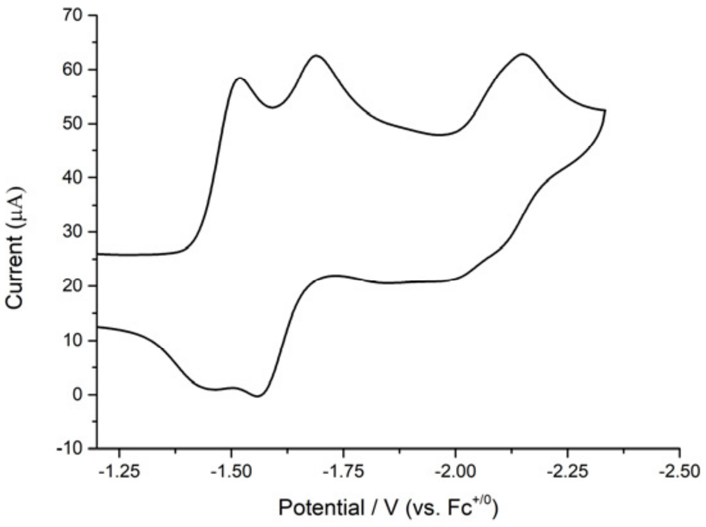

Figure S20. CV of **[Re(pmbpy)$(CO)_3(CH_3CN)$] (1- CH_3CN^+)** in Ar-saturated CH_3CN at 100 mV s^{-1}.

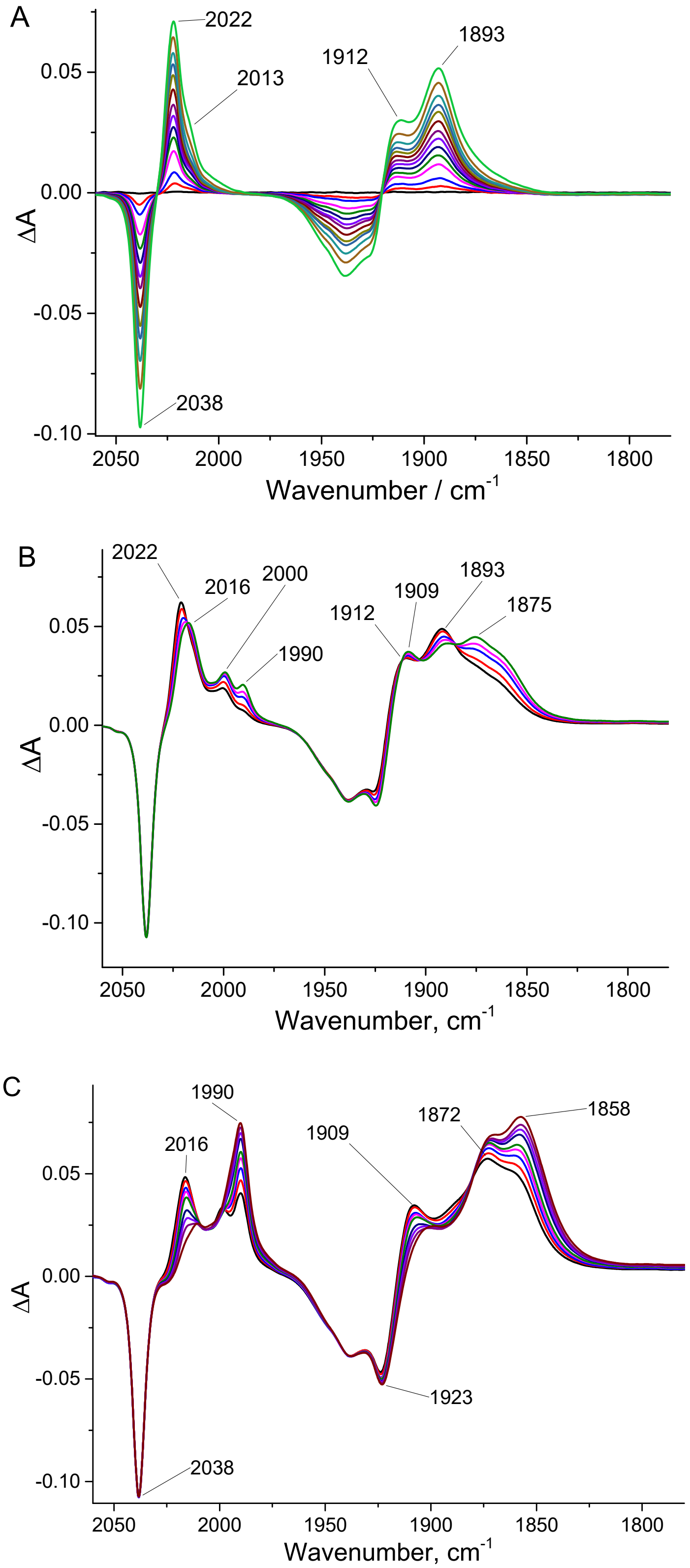

Figure S21. Difference IR spectra (expanded metal carbonyl region) measured during controlled potential electrolysis of **1-CH_3CN^+** in CD_3CN containing 0.1 M Bu_4NPF_6 under an N_2 atmosphere. Applied potentials: (A) −1.4 V vs $Fc^{+/0}$; (B) −1.6 V vs $Fc^{+/0}$ and (C) −1.7 V vs $Fc^{+/0}$.

VII. Cylic voltammetry of [(4,6-diphenyl-2,2′-bipyridine)Re(CO)$_3$Cl] (RediPh) under CO_2.

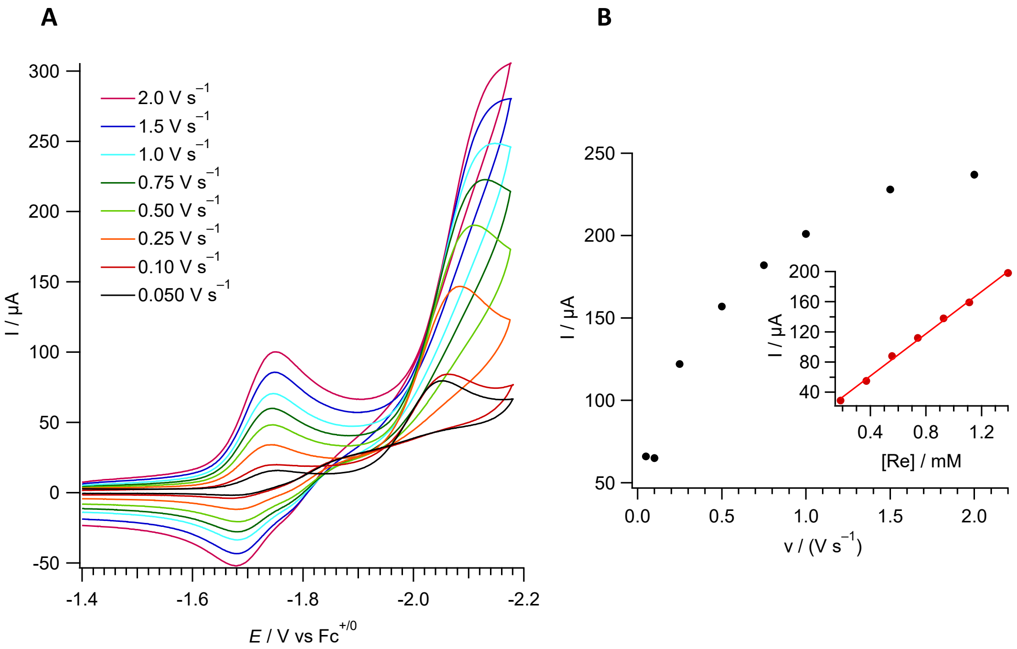

Figure S22. (A) Variable scan rate cyclic voltammograms of 1 mM [(4,6-diphenyl-2,2′-bipyridine)Re(CO)$_3$Cl] in CO_2-saturated CH_3CN with 0.1 M Bu_4NPF_6 at a glassy carbon electrode. (B) Catalytic current vs scan rate; inset: current dependence on catalyst concentration at 2 V s^{-1}.

VIII. DFT Calculations

DFT calculation were performed with Gaussian 16 and Gaussian 09. Solvent effects were taken in account by the conductor-like polarizable continuum model (CPCM) with acetonitrile as solvent. No constraints were imposed during geometry optimizations. The B3LYP functional, with the optimized def2-TZVP basis set for Re and Cl and the def2-SVP basis set for all other atoms were employed. The D3 version of Grimme's dispersion method was applied adopting the Becke-Johnson damping scheme. Gibbs free energies were determined using thermal corrections for entropy and enthalpy at 298 K to the electronic energies. In these calculations, the computed harmonic frequencies were scaled by 0.965 to account for anharmonicity.

Below are reported the computed scaled frequencies of the complexes under study, and the corresponding cartesian coordinates of the optimized structures.

#	Structure	Charge	Spin	Freq (cm^{-1})	Exp
**1-*cis***	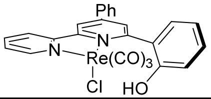	0	1	2022, 1921, 1904	2022, 1918, 1894
**1-*trans***	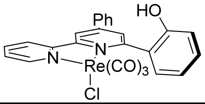	0	1	2018, 1917, 1895	
**^{TS}1'**		0	1	–28.8	
**^{TS}1''**		0	1	–35.0	
**[1-*cis*]$^{\bullet-}$**	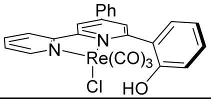	-1	2	2001, 1893, 1877	
**[1-*trans*]$^{\bullet-}$**		-1	2	1997, 1889, 1868	
**2-*cis***	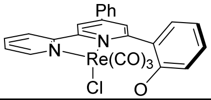	-1	1	2016, 1914, 1884	
**2 *trans***		-1	1	2010, 1912, 1887	
**1-CH_3CN^+**	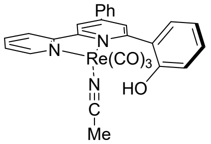	1	1	2036, 1946, 1929	
**1-$CH_3CN^\bullet$ *cis***	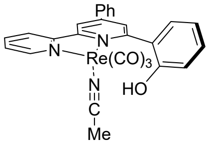	0	2	2014, 1918, 1899	
**1-$CH_3CN^\bullet$ *trans***		0	2	2014, 1917, 1899	
**1-PhO(CH_3CN)$^-$ *cis***	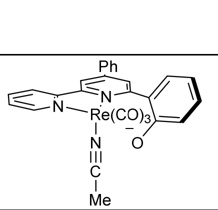	-1	2	2005, 1909, 1881	2000, 1909, 1876
**1-CH_3CN^- *cis***	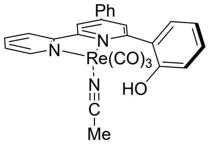	-1	1	1990, 1891, 1868	
**1-CH_3CN^- *trans***		-1	1	1985, 1887, 1864	

**1-OPh**	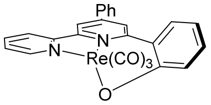	0	1	2011, 1903, 1891	2013, 1907, 1884
**1-OPh$^{\bullet-}$**	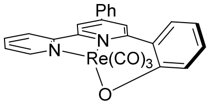	-1	2	1989, 1873, 1864	1990, 1872, 1858
**1-OPh^{2-}**	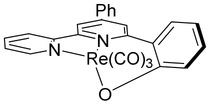	-2	1	1960, 1839, 1830	
**1-PhO^{2-}** ***cis***	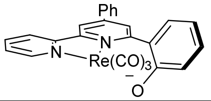	-2	1	1935, 1844, 1823	
**1-PhO^{2-}** ***trans***		-2	1	1937, 1848, 1823<br>609 (30)	
**1-PhO(CH$_3$CN)$^{2-}$** ***cis***	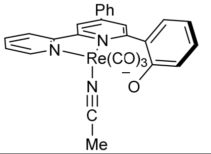	-2	1	1970, 1874, 1843	1995, 1877, 1862
**1-PhO(CH$_3$CN)$^{2-}$** ***trans***		-2	1	1972, 1878, 1848	
**1-PhO·CO$_2$$^{2-}$** ***cis***	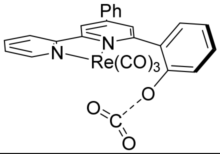	-2	1	2331, 1933, 1842, 1821	
**^{TS}1-PhO·CO$_2$$^{2-}$**		-2	1	-60	
**1-OCOOPh^{2-}**	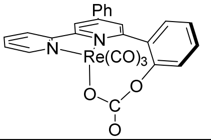	-2	1	1966, 1865, 1839, 1733	

1-*cis*

```
0 1
C 2.49337000 0.99725300 -0.11242700
C 3.18114300 -0.22227700 -0.10100100
C 2.39229000 -1.38033700 -0.08803600
C 1.00207900 -1.29387100 -0.09520900
C 1.09738300 1.03595300 -0.12277200
C 0.15180400 -2.50267300 -0.06655300
C 0.66981200 -3.80324900 -0.03370300
C -0.19960000 -4.88989100 0.00198900
H 0.19479500 -5.90696400 0.02797900
C -1.57484600 -4.65432100 0.00480600
C -2.02411200 -3.33924100 -0.03126200
H 3.03074000 1.94400100 -0.08620700
H -2.29675700 -5.47087500 0.03402000
H -3.08968100 -3.11363500 -0.03178700
N 0.35766300 -0.09484500 -0.12535400
N -1.18511100 -2.28921800 -0.06766100
Re -1.87392100 -0.19988100 -0.11322700
C -2.43054300 1.65754700 -0.09129300
O -2.85098200 2.73471900 -0.09055100
C -3.73982300 -0.64274500 0.01797600
O -4.85162600 -0.95991100 0.10902800
C -1.97590400 -0.18875600 -2.02684200
O -2.01512800 -0.17640300 -3.18638400
Cl -1.47873100 -0.27727200 2.38847100
H 1.74426000 -3.97222900 -0.03444800
H 2.87137700 -2.35561700 -0.09155200
C 4.65872200 -0.28732900 -0.09512600
C 5.32960900 -1.35038900 0.53769800
```

```
C 5.42178200 0.71541000 -0.72197600
C 6.72367800 -1.40676500 0.54535600
H 4.76020200 -2.12632300 1.05261300
C 6.81560100 0.65332900 -0.71945100
H 4.92267300 1.53716900 -1.23860400
C 7.47117400 -0.40657100 -0.08461600
H 7.22846300 -2.23302800 1.05064800
H 7.39241800 1.43398500 -1.22016300
H 8.56248800 -0.45288200 -0.08069800
C 0.45152200 2.37700400 -0.11200200
C 0.12782300 2.99317200 1.11529600
C 0.31088300 3.09717800 -1.30707500
C -0.29057400 4.33073400 1.12047700
C -0.13451600 4.41858200 -1.29704400
C -0.41868900 5.03712900 -0.07420700
H -0.52982100 4.79121400 2.08098600
H -0.24670800 4.96620400 -2.23479800
H -0.75593000 6.07615200 -0.05154400
O 0.23720700 2.34837500 2.30361400
H -0.09517800 1.42530800 2.24873800
H 0.56513000 2.60713100 -2.24969300
```

1-*trans*

```
0 1
C -2.49314500 1.08406600 -0.00578200
C -3.19846300 -0.12495400 0.03807700
C -2.43103800 -1.28776900 0.18518500
C -1.04048600 -1.21685100 0.24240600
C -1.09857400 1.10669700 0.06891200
C -0.21283900 -2.42954400 0.41682700
C -0.75377000 -3.69617200 0.67107200
C 0.09603400 -4.78432000 0.84822900
H -0.31562800 -5.77502100 1.04784400
C 1.47524900 -4.58404600 0.77154100
C 1.94761200 -3.30191100 0.51672900
H -3.01600800 2.03417700 -0.10523900
H 2.18186700 -5.40345700 0.90584300
H 3.01637000 -3.10287800 0.44536900
N -0.37509600 -0.03409100 0.15438500
N 1.12717100 -2.25081900 0.34252200
Re 1.83332400 -0.23710600 -0.17240800
C 2.33928000 1.54085900 -0.75821100
O 2.70663400 2.56732500 -1.14405400
C 3.63628300 -0.77886600 -0.54832300
O 4.70985300 -1.15653100 -0.77479700
C 2.33326200 0.17938500 1.62991200
O 2.66040900 0.42292400 2.71790400
Cl 1.05195400 -0.85139000 -2.50389100
H -1.83063400 -3.83434200 0.74035600
H -2.92550100 -2.25215000 0.26569800
C -4.67456900 -0.17461200 -0.04813600
C -5.32229000 -1.28275900 -0.62532500
C -5.45944600 0.88469900 0.44414200
C -6.71423900 -1.32805700 -0.71035000
H -4.73350500 -2.10518900 -1.03579300
C -6.85158000 0.83445700 0.36464600
H -4.98050100 1.74367300 0.91735400
C -7.48366000 -0.27079600 -0.21414200
H -7.19992100 -2.19094800 -1.17126300
H -7.44580800 1.66038400 0.76172600
H -8.57345500 -0.30813700 -0.27821600
C -0.41828100 2.42631400 0.06506700
C -0.48324800 3.26521800 -1.05453300
C 0.21566500 2.88932200 1.23617300
C 0.11366800 4.52651800 -1.04130700
C 0.82166500 4.15103700 1.24832700
C 0.77207300 4.96108900 0.11268500
H 0.07212000 5.16204700 -1.92767900
H 1.32019000 4.49670100 2.15776300
H 1.24935100 5.94347500 0.13557600
O 0.18366000 2.08013900 2.32155900
H 0.74949400 2.43237300 3.02345400
```

H -0.99091000 2.90535600 -1.95219300

^{TS}1'

0 1
C 2.47773500 0.75309500 0.08060000
C 2.92545200 -0.56279800 -0.05574900
C 2.00143100 -1.48751200 -0.55616200
C 0.67357000 -1.10445700 -0.71362500
C 1.15864700 1.12580000 -0.22403100
C -0.32715500 -2.07295600 -1.21814400
C 0.02173800 -3.19253500 -1.98264700
C -0.98117500 -4.02812000 -2.46763000
H -0.72414400 -4.90226700 -3.06835800
C -2.31450500 -3.72384200 -2.18389100
C -2.59126000 -2.59689200 -1.41943100
H 3.16685600 1.51986300 0.41494600
H -3.13330900 -4.34532700 -2.54728500
H -3.61565700 -2.32247000 -1.16691500
N 0.22410100 0.15322000 -0.44855800
N -1.62085000 -1.79458100 -0.94702400
Re -1.94360300 -0.11861600 0.41252900
C -2.03570300 1.26598100 1.76913300
O -2.08267900 2.05324700 2.61455400
C -3.62724000 -0.75154900 1.05628900
O -4.64138900 -1.17633700 1.42333800
C -2.84097700 0.93834800 -0.91286600
O -3.38836300 1.57354600 -1.71544200
Cl -0.70429900 -1.55850800 2.08551800
H 1.06562300 -3.39567800 -2.21530200
H 2.30376100 -2.50781800 -0.78051900
C 4.31899600 -0.94032300 0.26300900
C 4.62539700 -2.23460000 0.72230400
C 5.36501700 -0.01053300 0.11128200
C 5.94069500 -2.58702500 1.02740900
H 3.82568700 -2.96361900 0.86717100
C 6.68032100 -0.36738100 0.41041300
H 5.15002400 0.98888100 -0.27138200
C 6.97227200 -1.65531600 0.87157500
H 6.16047800 -3.59225700 1.39381000
H 7.48228700 0.36211100 0.27708800
H 8.00200200 -1.93274600 1.10815300
C 0.85266000 2.57444600 -0.39922500
C 1.85726700 3.47646800 -0.86333300
C -0.43444000 3.11348400 -0.23427300
C 1.55189600 4.82882500 -1.08133500
C -0.73930600 4.45266000 -0.45195700
C 0.26850700 5.32324300 -0.87339100
H 2.34873100 5.49115100 -1.43097900
H -1.75904000 4.80831000 -0.29453000
H 0.05917900 6.38066000 -1.04934200
O 3.10974000 3.03320000 -1.13223000
H 3.63756400 3.75647800 -1.50098300
H -1.22698200 2.44677300 0.07229500

^{TS}1"

0 1
C -2.49175300 0.88045500 0.00287800
C -2.95250000 -0.42533400 0.19192500
C -2.05726000 -1.31591700 0.79514900
C -0.73131900 -0.92774800 0.96177200
C -1.18813800 1.27258500 0.36711200
C 0.25309700 -1.91493100 1.46690200
C -0.08962900 -2.90103600 2.39890500
C 0.88898400 -3.78571000 2.84543300
H 0.64132000 -4.55643700 3.57743900
C 2.18922100 -3.66368000 2.34914000
C 2.45973000 -2.65997900 1.42704700
H -3.15879800 1.62345600 -0.42734700
H 2.98853300 -4.33241900 2.66976000
H 3.45817900 -2.52857000 1.00930000
N -0.26361200 0.31191500 0.66365100

N 1.51321000 -1.80554600 0.99678100
Re 1.85046100 -0.23943500 -0.50157600
C 1.94019900 1.15586400 -1.84720200
O 1.98231900 1.96296400 -2.67315700
C 3.33220400 -1.08384800 -1.31912200
O 4.23033600 -1.64354300 -1.80068100
C 3.02155100 0.71940200 0.67084800
O 3.73973000 1.29348000 1.37967600
Cl 0.22760600 -1.52221800 -1.96768100
H -1.10682300 -2.95667900 2.78468200
H -2.36345900 -2.32708400 1.05506400
C -4.31581400 -0.84114200 -0.20415800
C -4.56378400 -2.16570600 -0.61095100
C -5.38704000 0.07146000 -0.19102200
C -5.84422300 -2.56407400 -0.99653700
H -3.74245500 -2.88352900 -0.65276900
C -6.66830000 -0.33025400 -0.57081300
H -5.22604900 1.09760400 0.14352300
C -6.90117700 -1.64837200 -0.97625900
H -6.01598100 -3.59305100 -1.32014600
H -7.49011200 0.38872600 -0.54438500
H -7.90403900 -1.96122300 -1.27564000
C -0.97780800 2.74063200 0.57117200
C 0.22823400 3.47802600 0.48687100
C -2.12144200 3.47403000 0.97792700
C 0.24876400 4.86089200 0.73321500
C -2.10327500 4.84089200 1.23156900
C -0.90418900 5.54610600 1.09638100
H 1.20105400 5.38960800 0.63704300
H -3.01712700 5.34605600 1.54875900
H -0.86111400 6.62046300 1.28788000
O 1.36801800 2.84633300 0.16382900
H 2.10356300 3.47341400 0.10767700
H -3.05699000 2.94085100 1.13845400

[1-*cis*]$^{\bullet-}$

-1 2
C 2.48946100 1.00335800 -0.08640400
C 3.18308600 -0.24683200 -0.05636900
C 2.39161400 -1.38302900 -0.01076300
C 0.98132100 -1.31199500 -0.00985900
C 1.10586500 1.04380000 -0.07310400
C 0.15464600 -2.48275700 0.01614400
C 0.65241800 -3.81344100 0.09794200
C -0.21276500 -4.88509100 0.12016900
H 0.17583400 -5.90359900 0.18607000
C -1.60880200 -4.64803700 0.05958400
C -2.04257000 -3.33812500 -0.02054200
H 3.03209600 1.94673200 -0.06956800
H -2.33266600 -5.46314500 0.07598800
H -3.10828600 -3.11091500 -0.06967100
N 0.33226200 -0.07561300 -0.05518300
N -1.20939200 -2.27610200 -0.04355400
Re -1.86542600 -0.19855500 -0.16123400
C -2.41098100 1.66280200 -0.17170900
O -2.83229000 2.74385500 -0.19912300
C -3.73835600 -0.64092500 -0.12791900
O -4.85855100 -0.95522700 -0.10032300
C -1.86110300 -0.18866200 -2.06577900
O -1.83469200 -0.18099700 -3.22982200
Cl -1.65372000 -0.27978900 2.40791700
H 1.72650700 -3.98555600 0.14992000
H 2.86166400 -2.36430200 -0.01007400
C 4.65731200 -0.31492000 -0.08014900
C 5.35246700 -1.42025300 0.45561500
C 5.42127700 0.73084400 -0.63994700
C 6.74518400 -1.48139200 0.42324100
H 4.79578600 -2.23286900 0.92620100
C 6.81558800 0.66896500 -0.67185600
H 4.91686900 1.59334300 -1.07885900
C 7.48739500 -0.43685200 -0.14127300
H 7.25675800 -2.34679300 0.85186500

```
H 7.38115200 1.48990800 -1.11975800
H 8.57860800 -0.48376500 -0.16343600
C 0.47602500 2.39546700 -0.05254500
C 0.07365400 2.97912200 1.16778100
C 0.42761100 3.16428200 -1.22376600
C -0.32434300 4.32308100 1.19298100
C 0.00212000 4.49365900 -1.20080600
C -0.35904200 5.07503000 0.01918700
H -0.62044300 4.75386300 2.15178700
H -0.03375000 5.07501900 -2.12463700
H -0.68104500 6.11879100 0.05641200
O 0.09132100 2.29976100 2.34189200
H -0.21311300 1.37228400 2.22954700
H 0.74333900 2.70333000 -2.16277500
```

[1-*trans*]•–

```
-1 2
C -2.48779000 1.08371600 -0.04672200
C -3.20662900 -0.15168900 0.00320800
C -2.43944100 -1.29819400 0.13712100
C -1.02915800 -1.25184300 0.18596500
C -1.10445600 1.09752900 0.00693100
C -0.22882500 -2.43063300 0.34643300
C -0.75553900 -3.73877900 0.53594900
C 0.08641700 -4.81626100 0.70305600
H -0.32483000 -5.81701500 0.85197100
C 1.48868900 -4.60769900 0.68441400
C 1.95106200 -3.31975100 0.48965900
H -3.00909900 2.03526200 -0.13334300
H 2.19473500 -5.42830500 0.81418200
H 3.02222700 -3.11440800 0.46090500
N -0.35305300 -0.03564500 0.08175900
N 1.14093100 -2.25340300 0.31910300
Re 1.83762300 -0.23383000 -0.12218700
C 2.36620100 1.54409200 -0.68495200
O 2.76252400 2.57127600 -1.05229700
C 3.66376900 -0.76784300 -0.39270200
O 4.75522200 -1.14038000 -0.55493600
C 2.23102300 0.19702000 1.69046500
O 2.49625800 0.45580700 2.79619000
Cl 1.22594100 -0.84975300 -2.55620200
H -1.83419300 -3.88830800 0.55626700
H -2.93102200 -2.26461200 0.22850700
C -4.68080000 -0.19614800 -0.05328900
C -5.36458800 -1.34660600 -0.50263800
C -5.45778600 0.91491500 0.33815900
C -6.75765200 -1.38770000 -0.54736100
H -4.79633900 -2.21353500 -0.84500200
C -6.85244300 0.87325500 0.29249600
H -4.96549300 1.81612600 0.70722500
C -7.51271500 -0.27753700 -0.14968400
H -7.25882200 -2.29022000 -0.90614100
H -7.42787500 1.74640300 0.61032200
H -8.60403500 -0.30886900 -0.18818900
C -0.43109500 2.42574500 -0.00247500
C -0.44353500 3.23216300 -1.14626600
C 0.14635900 2.93270100 1.17959600
C 0.13842300 4.50257700 -1.14436900
C 0.74011800 4.20031400 1.18365000
C 0.73704500 4.97929500 0.02408700
H 0.13194300 5.11086600 -2.05111600
H 1.19174300 4.57683800 2.10574300
H 1.20364400 5.96722600 0.04083300
O 0.07475200 2.15899500 2.29175900
H 0.64449800 2.51822800 2.98636500
H -0.90491100 2.83929000 -2.05518100
```

2-*cis*

```
-1 1
C 2.46407800 0.93611300 -0.18626800
C 3.09075900 -0.30732900 -0.19291800
```

C 2.26617900 -1.43509200 -0.37070700
C 0.88935100 -1.27938900 -0.45584000
C 1.06965500 1.05901400 -0.34774800
C -0.01130100 -2.44184300 -0.59794000
C 0.43780400 -3.72571700 -0.93712000
C -0.47932200 -4.76328600 -1.06882500
H -0.13986900 -5.76546800 -1.33648600
C -1.83580700 -4.49935700 -0.86108200
C -2.21636900 -3.20575800 -0.52808200
H 3.02550000 1.85570300 -0.04441700
H -2.59234400 -5.27890500 -0.95529600
H -3.26232000 -2.95430600 -0.35295100
N 0.28990000 -0.05565500 -0.39358000
N -1.32949800 -2.20195800 -0.39915000
Re -1.86584400 -0.16679400 0.21660200
C -2.13775700 1.64883100 0.85031700
O -2.33145200 2.70707400 1.27320600
C -3.61135200 -0.61788400 0.87267000
O -4.65492800 -0.94390600 1.26596200
C -2.61192800 0.26903500 -1.48651000
O -3.07288100 0.52640600 -2.52360800
Cl -0.80073700 -0.83013000 2.43153500
H 1.49640600 -3.90854400 -1.11177600
H 2.70536700 -2.42794400 -0.42792200
C 4.55890700 -0.44107300 -0.04423300
C 5.12222100 -1.58961000 0.54287800
C 5.42376300 0.58007800 -0.48078000
C 6.50477900 -1.71148200 0.69129000
H 4.47293700 -2.38620500 0.91105200
C 6.80620700 0.45543100 -0.33651200
H 5.01200900 1.47098500 -0.95799800
C 7.35267000 -0.69033300 0.25082000
H 6.92131500 -2.60675200 1.15861800
H 7.46069000 1.25534700 -0.69036500
H 8.43483600 -0.78702100 0.36451100
C 0.48638000 2.39037800 -0.51139900
C 0.92344000 3.47368800 0.36451800
C -0.43430300 2.62410900 -1.55005300
C 0.29861400 4.75020800 0.09496800
C -0.99332400 3.87658600 -1.77443700
C -0.61855700 4.93856800 -0.92336200
H 0.59269200 5.58002200 0.74533800
H -1.69468700 4.03473300 -2.59656000
H -1.05206800 5.93254200 -1.07901200
O 1.76764700 3.31673000 1.29373700
H -0.68823100 1.79712500 -2.21514000

2-*trans*

-1 1
C -2.47932900 1.07394000 0.04240500
C -3.17056900 -0.13853400 0.08915900
C -2.39855800 -1.29575100 0.28121100
C -1.01214800 -1.20313200 0.35223900
C -1.08007600 1.13140000 0.15762800
C -0.16723300 -2.40228000 0.53488400
C -0.68109700 -3.66299300 0.86694000
C 0.18766000 -4.73558200 1.04351600
H -0.20221500 -5.72072900 1.30544900
C 1.56007200 -4.52762800 0.88666700
C 2.00513400 -3.25362800 0.55622900
H -3.01595000 2.01478500 -0.07259100
H 2.28062900 -5.33557700 1.01702800
H 3.06626000 -3.04664100 0.41882200
N -0.35706200 -0.01583500 0.25033100
N 1.16570300 -2.21659400 0.38403900
Re 1.81264400 -0.21176500 -0.22898200
C 2.22076800 1.57251100 -0.87584700
O 2.53075100 2.59393100 -1.32303400
C 3.56218200 -0.75603100 -0.78093300
O 4.60592500 -1.14639600 -1.11719200
C 2.51726500 0.19414900 1.51105700
O 3.03094800 0.36378600 2.53793000

Cl 0.83380200 -0.86919800 -2.49367200
H -1.75202100 -3.80357700 0.99932300
H -2.88503000 -2.26345200 0.37413400
C -4.64529500 -0.20625600 -0.03171200
C -5.26613000 -1.32493600 -0.61820700
C -5.45694400 0.84525000 0.43295200
C -6.65509300 -1.38796800 -0.73974900
H -4.65647600 -2.14306200 -1.00652100
C -6.84609600 0.77876900 0.31584600
H -4.99983400 1.71244400 0.91288000
C -7.45057600 -0.33713800 -0.27197700
H -7.11782800 -2.26012100 -1.20721700
H -7.46002900 1.60053600 0.69145900
H -8.53784200 -0.38783500 -0.36472600
C -0.41781200 2.44176000 0.20260100
C -0.65974900 3.38509000 -0.80846500
C 0.35487400 2.78294800 1.39245600
C -0.10676100 4.66337100 -0.76877600
C 0.90413600 4.12005700 1.37947500
C 0.68454100 5.01360900 0.34435800
H -0.27595600 5.37145100 -1.58302500
H 1.50721500 4.40446700 2.24778100
H 1.13106200 6.01291400 0.39406300
O 0.50569200 1.99084600 2.36502100
H -1.27092200 3.08680900 -1.66686500

1-CH_3CN^+

1 1
C 2.53086400 1.05534800 -0.06192700
C 3.23681300 -0.15596800 -0.04602300
C 2.46399400 -1.32320800 0.01661800
C 1.07257200 -1.25628000 0.02737500
C 1.13558600 1.07592800 -0.05331000
C 0.24134600 -2.47660600 0.10236600
C 0.77153100 -3.74625400 0.35727700
C -0.08034400 -4.84602900 0.42416500
H 0.32240000 -5.84008300 0.62471700
C -1.44886300 -4.65257000 0.23627200
C -1.91224500 -3.36467300 -0.00894400
H 3.05534100 2.00953400 -0.05028500
H -2.15638600 -5.48086900 0.28016200
H -2.97383600 -3.17342900 -0.15972700
N 0.40864600 -0.06957200 -0.04061600
N -1.09043800 -2.30220800 -0.07426900
Re -1.79982100 -0.24796200 -0.40952700
C -2.38183800 1.59901300 -0.59171300
O -2.81257900 2.66251700 -0.71251300
C -3.63867200 -0.74705300 -0.69086200
O -4.73153900 -1.09269400 -0.85193000
C -1.46504000 -0.32442100 -2.32120100
O -1.24591100 -0.36314800 -3.45360100
H 1.83965500 -3.87814900 0.51646600
H 2.95312500 -2.29368900 0.02111100
C 4.71350000 -0.19994200 -0.07854500
C 5.41694900 -1.24797300 0.54445100
C 5.44365800 0.80848300 -0.73534800
C 6.81110700 -1.28391100 0.51330900
H 4.87452500 -2.02751400 1.08230600
C 6.83744900 0.76599100 -0.77204400
H 4.91891700 1.61820200 -1.24544100
C 7.52557300 -0.27877400 -0.14653500
H 7.34212500 -2.09795600 1.01130400
H 7.38864000 1.54984200 -1.29590100
H 8.61707800 -0.30951600 -0.17311400
C 0.45275000 2.39689000 -0.00591200
C -0.05420400 2.88141600 1.21753900
C 0.40981300 3.22028000 -1.14104400
C -0.62642800 4.15776300 1.27644900
C -0.15998400 4.49038800 -1.08102100
C -0.68029400 4.95274000 0.13354800
H -1.01607800 4.50972000 2.23337100
H -0.20072100 5.11535900 -1.97484000

H -1.13012900 5.94639000 0.19321200
O -0.00449200 2.16085500 2.36825300
H 0.49010600 1.33929200 2.23633000
H 0.82046600 2.84148800 -2.07949300
C -2.03537800 -0.07347100 2.85596100
N -1.99725100 -0.18272500 1.70714800
C -2.05813300 0.11021300 4.29246900
H -1.59240400 -0.75315900 4.78967800
H -1.49358400 1.02301100 4.53547000
H -3.09570300 0.21604700 4.64148000

1-$CH_3CN^{\bullet}$-*cis*

0 2
C 2.51617600 1.05600800 -0.10472400
C 3.22960500 -0.18448300 -0.04468400
C 2.45374700 -1.32893900 0.04554800
C 1.04297200 -1.28045800 0.04396200
C 1.13476600 1.07755700 -0.09719500
C 0.23453400 -2.46304000 0.10993000
C 0.74776800 -3.77123700 0.32843700
C -0.10100600 -4.85474900 0.39018200
H 0.29846800 -5.85592500 0.56478200
C -1.49412000 -4.65206200 0.23265500
C -1.94443300 -3.36310200 0.02007100
H 3.04478600 2.00756400 -0.10038200
H -2.20441600 -5.47792000 0.27581000
H -3.00880700 -3.16354500 -0.10947200
N 0.37279200 -0.05712500 -0.05192300
N -1.12643500 -2.29000700 -0.04584800
Re -1.80542300 -0.24581800 -0.40261600
C -2.37762000 1.60305700 -0.58981100
O -2.81097000 2.67030800 -0.70886200
C -3.65189200 -0.74476000 -0.64441800
O -4.75164900 -1.09006000 -0.79024100
C -1.49323900 -0.34182800 -2.30711400
O -1.28108700 -0.39251400 -3.44422500
H 1.81890000 -3.91458600 0.46308000
H 2.93680900 -2.30349400 0.07519100
C 4.70369100 -0.23016100 -0.07690000
C 5.41986500 -1.31060200 0.48156700
C 5.44639000 0.81316000 -0.66894500
C 6.81299000 -1.35015600 0.44034800
H 4.88028200 -2.12000800 0.97683200
C 6.84115900 0.77259000 -0.70968500
H 4.92560100 1.65609400 -1.12634600
C 7.53407300 -0.30860800 -0.15612600
H 7.34152800 -2.19604500 0.88681200
H 7.39029200 1.59053000 -1.18264600
H 8.62566400 -0.33887600 -0.18540800
C 0.46439200 2.40787200 -0.05221000
C -0.02085600 2.89296900 1.17965400
C 0.40813400 3.23885400 -1.17845400
C -0.57191800 4.17642200 1.26222500
C -0.14201800 4.51923300 -1.10053600
C -0.63289600 4.98206100 0.12500800
H -0.94046200 4.52735500 2.22821700
H -0.18924000 5.15129300 -1.98955400
H -1.06653100 5.98225600 0.19924300
O 0.04682100 2.15228800 2.31798100
H 0.45556100 1.29565200 2.11996100
H 0.79851500 2.86031900 -2.12586800
C -1.98375600 -0.03747700 2.88041300
N -1.97222600 -0.15705800 1.73201000
C -1.96946800 0.16305100 4.31597200
H -1.49694200 -0.69682100 4.81279200
H -1.39279100 1.07454800 4.53389000
H -2.99654100 0.28007900 4.69137600

1-$CH_3CN^{\bullet}$-*trans*

0 2
C 2.50903300 0.97019600 0.20529800

```
C 3.19438600 -0.28454900 0.11015500
C 2.39308400 -1.41316100 0.05818500
C 0.98323700 -1.32982900 0.05384000
C 1.12552500 1.02100500 0.20892900
C 0.14351700 -2.49030500 -0.00356900
C 0.61898200 -3.82794400 0.08022300
C -0.26153600 -4.88694200 0.03977600
H 0.10860900 -5.91179200 0.11145600
C -1.64871800 -4.62921800 -0.08699900
C -2.06136600 -3.31286700 -0.17064400
H 3.06059000 1.89847200 0.34748100
H -2.38299200 -5.43430800 -0.12020900
H -3.11961500 -3.07040100 -0.27557000
N 0.34414200 -0.08771600 0.08241600
N -1.21210600 -2.26336500 -0.13638000
Re -1.82203000 -0.17424900 -0.33618400
C -2.32614700 1.70095800 -0.42410000
O -2.71526300 2.78841900 -0.50976600
C -3.68010400 -0.59262400 -0.63991200
O -4.78816800 -0.89069600 -0.82242800
C -1.48089900 -0.16066100 -2.23728900
O -1.24863100 -0.15029800 -3.37163600
H 1.68601800 -4.01465200 0.19267800
H 2.85369300 -2.39546600 -0.02669900
C 4.66706000 -0.35750200 0.07209300
C 5.35925300 -1.51308800 0.49322800
C 5.43204800 0.73466900 -0.38892900
C 6.75127900 -1.57651200 0.44585000
H 4.80169300 -2.36551200 0.88588500
C 6.82574000 0.67002000 -0.43604000
H 4.92928700 1.63765700 -0.73961100
C 7.49486500 -0.48511400 -0.01961000
H 7.26142400 -2.48154900 0.78476000
H 7.39267500 1.52782300 -0.80620700
H 8.58561900 -0.53453600 -0.05363600
C 0.48511800 2.35606600 0.37908500
C 0.54591300 3.29554900 -0.66867600
C -0.10563400 2.72764500 1.59497900
C 0.01638600 4.58021200 -0.48932800
C -0.63400600 4.00537200 1.77628700
C -0.56935900 4.92934200 0.72641900
H 0.06958300 5.28829000 -1.31869700
H -1.09141100 4.28040500 2.72877900
H -0.98044400 5.93362400 0.85479000
O 1.09176400 2.99004300 -1.87329800
H 1.49264900 2.10620300 -1.82376600
H -0.14039100 1.99568100 2.40397800
C -2.04742000 -0.33283200 2.95043100
N -2.01183400 -0.25706000 1.79864000
C -2.08745100 -0.41750800 4.39797300
H -1.48372100 -1.27229300 4.73671400
H -1.68116100 0.50715500 4.83420900
H -3.12561500 -0.55119800 4.73584400
```

1-PhO(CH_3CN)$^-$-*cis*

```
-1 2
C   2.46893800    1.00456900   -0.20099600
C   3.17865800   -0.23077300   -0.14542800
C   2.41082000   -1.38122600   -0.07222700
C   0.99786800   -1.32810300   -0.07146100
C   1.08002600    1.03531100   -0.19680700
C   0.18782700   -2.50497600    0.02424300
C   0.70281000   -3.82643700    0.15723700
C  -0.14570500   -4.90561200    0.25626400
H   0.25809400   -5.91507000    0.36012900
C  -1.54923400   -4.68790900    0.22426600
C  -2.00161900   -3.38981000    0.09015200
H   2.99943900    1.95517200   -0.19930600
H  -2.26166000   -5.50969000    0.30075100
H  -3.07155400   -3.17900900    0.05644600
N   0.33576700   -0.10401700   -0.16633900
N  -1.18487900   -2.31806300   -0.00988800
```

```
Re -1.86829100   -0.26220700   -0.26291500
C  -2.43221400    1.60053000   -0.37055200
O  -2.89557000    2.65970600   -0.43264000
C  -3.73204500   -0.73454000   -0.22462700
O  -4.84721500   -1.07059500   -0.19608200
C  -1.84183100   -0.36206500   -2.18546800
O  -1.79527100   -0.40737700   -3.34460900
H   1.78037600   -3.98270500    0.18642200
H   2.89754600   -2.35399400   -0.04453800
C   4.65599200   -0.27727200   -0.17085300
C   5.36934000   -1.34578200    0.41187900
C   5.40112300    0.75392800   -0.77946500
C   6.76315500   -1.38636600    0.37839800
H   4.82560700   -2.14493400    0.91932700
C   6.79637200    0.71313600   -0.81298800
H   4.87989600    1.58718000   -1.25388900
C   7.48691100   -0.35676100   -0.23495500
H   7.28968000   -2.22337000    0.84382000
H   7.34784100    1.52185700   -1.29914500
H   8.57872600   -0.38729700   -0.25843000
C   0.42335300    2.37010200   -0.20604400
C   0.16762100    3.00452900    1.07453500
C   0.20810600    3.04011800   -1.41280800
C  -0.34317700    4.34717900    0.98329500
C  -0.29751700    4.34459000   -1.45158800
C  -0.56622700    4.98603000   -0.23150600
H  -0.54802300    4.86149700    1.92844600
H  -0.47526600    4.84825300   -2.40479400
H  -0.95764100    6.00971000   -0.23400300
O   0.39498900    2.41886300    2.18385700
H   0.43872200    2.51740800   -2.34761100
C  -1.31900600    0.22597200    2.93168600
N  -1.65074200   -0.08918800    1.87095200
C  -0.88770600    0.69039100    4.23425900
H  -0.14945500   -0.00524800    4.65957000
H  -0.41844000    1.67094400    4.04917200
H  -1.74438600    0.77634000    4.91833300
```

1-CH_3CN^--*cis*

```
-1 1
C 2.48862900 1.10480400 -0.00340600
C 3.21788200 -0.15033700 0.17111500
C 2.42627100 -1.25665000 0.43693400
C 1.01511400 -1.21557100 0.46435400
C 1.12648100 1.15123000 0.08596500
C 0.22521100 -2.37930200 0.52092700
C 0.68878900 -3.68023700 0.91458900
C -0.16242500 -4.75347300 0.95318900
H 0.20261000 -5.73302200 1.27269000
C -1.53592500 -4.57602200 0.59085800
C -1.94036900 -3.31552100 0.21005100
H 3.02476900 2.05080800 -0.06784000
H -2.24887100 -5.40091300 0.61054200
H -2.97750100 -3.13461000 -0.08190800
N 0.31492700 0.03308300 0.30000800
N -1.12120500 -2.23835000 0.15576500
Re -1.68065100 -0.27650700 -0.59277900
C -2.15979500 1.54208500 -1.08124100
O -2.51353700 2.60722200 -1.38336800
C -3.37658700 -0.86674700 -1.30490300
O -4.37015500 -1.26629800 -1.76738000
C -0.82481000 -0.51679500 -2.29706400
O -0.28312300 -0.65720600 -3.31495800
H 1.73028000 -3.79987200 1.21617900
H 2.89454700 -2.23498400 0.54636300
C 4.67121700 -0.21633600 0.06271100
C 5.42086400 -1.33866200 0.51083800
C 5.41889400 0.85321700 -0.49998100
C 6.80610700 -1.39030500 0.39266600
H 4.90459200 -2.17930800 0.97870100
C 6.80862200 0.79820800 -0.61310800
```

```
H 4.89668400 1.73352400 -0.87757400
C 7.52218400 -0.32152300 -0.17075100
H 7.34075800 -2.27318400 0.75506600
H 7.34163900 1.64297400 -1.05936700
H 8.61041800 -0.36228700 -0.25734500
C 0.46025500 2.47359800 0.17595500
C -0.30443200 2.75352000 1.33552600
C 0.64226800 3.48896500 -0.77022500
C -0.86950100 4.01973300 1.51833900
C 0.07967500 4.75644500 -0.58995700
C -0.67563700 5.01749100 0.55755200
H -1.45147200 4.20907100 2.42328200
H 0.22568300 5.53384400 -1.34344700
H -1.12293100 6.00343100 0.70826800
O -0.46031600 1.80746300 2.29172800
H -0.22705700 0.94405800 1.86056700
H 1.23151600 3.26741500 -1.66340900
C -2.87712100 0.12400500 2.47401200
N -2.51358800 -0.03918900 1.39045700
C -3.28121500 0.37859000 3.84319000
H -3.25135800 -0.55155600 4.42913400
H -2.58225100 1.10825600 4.27906100
H -4.30097700 0.78965800 3.86883200
```

1-CH_3CN^-*trans*

```
-1 1
C 2.50174800 0.94587800 0.25505200
C 3.18518700 -0.34550400 0.18599200
C 2.36030600 -1.45114000 0.17626100
C 0.94265000 -1.36444500 0.17629700
C 1.12183100 1.00766900 0.27441900
C 0.10422300 -2.48666900 0.12948600
C 0.53443300 -3.85497100 0.25345400
C -0.36240100 -4.88789600 0.21363500
H -0.01781500 -5.91972500 0.32225800
C -1.75828600 -4.60319900 0.04161400
C -2.13108100 -3.28379000 -0.08365000
H 3.06129900 1.85653700 0.46920900
H -2.50885600 -5.39359800 0.00672700
H -3.18265800 -3.02281600 -0.22470500
N 0.30368100 -0.07800800 0.16015900
N -1.26961200 -2.23836300 -0.05322900
Re -1.80231600 -0.16386200 -0.39614100
C -2.24521000 1.71769400 -0.60516000
O -2.59403100 2.81359000 -0.77809100
C -3.65080100 -0.56746600 -0.78500800
O -4.75501500 -0.85236400 -1.03408600
C -1.35456800 -0.24791900 -2.26017900
O -1.05891300 -0.30083300 -3.38303700
H 1.59473100 -4.06280000 0.40369400
H 2.79870400 -2.44690000 0.11050900
C 4.64286500 -0.43488800 0.11412200
C 5.34712300 -1.64465600 0.35362600
C 5.43057800 0.70454200 -0.19602300
C 6.73459600 -1.71132100 0.27618000
H 4.79345300 -2.54598400 0.62426600
C 6.82298200 0.63375600 -0.27189700
H 4.93882900 1.65522700 -0.40712300
C 7.49314700 -0.57130500 -0.03752100
H 7.23683700 -2.66280200 0.47301400
H 7.39135400 1.53433700 -0.52196600
H 8.58292000 -0.62486000 -0.09254300
C 0.51253700 2.36053800 0.43230800
C 0.73385800 3.33404200 -0.56509200
C -0.22463700 2.71600400 1.56915400
C 0.19552400 4.62043300 -0.42623500
C -0.75003900 4.00034100 1.71938100
C -0.54275000 4.94866800 0.71099000
H 0.37144100 5.35058600 -1.21922100
H -1.32084800 4.25949400 2.61386100
H -0.95480600 5.95605400 0.81199800
O 1.45590100 3.04614200 -1.67776600
```

H 1.88393200 2.17803800 -1.51843100
H -0.38210500 1.96286200 2.34256000
C -2.27537700 -0.19338500 2.89856900
N -2.15392100 -0.14518200 1.75084300
C -2.41793000 -0.24581000 4.34258300
H -2.17309800 -1.25489000 4.70560700
H -1.73522400 0.47978000 4.80924200
H -3.45203800 -0.00149500 4.62686000

1-OPh

0 1
Re 1.83788700 -0.07937500 0.29549200
N -0.32248100 -0.02602800 0.00105600
C -0.04158100 -2.33997700 -0.48636200
O 1.54220500 1.13232600 -1.45521800
C -0.94405200 -1.22358000 -0.12919900
C -1.02642900 1.12432100 -0.10800300
C -0.86055100 3.62027800 -0.18291000
C -2.32815200 -1.32507000 -0.07463900
H -2.80927600 -2.29933100 -0.12670600
C 0.90055600 2.25994000 -1.24135100
C -0.29884200 2.35405400 -0.44620000
C -2.43073300 1.07203600 -0.05202600
H -2.99716500 1.99645200 -0.14637500
C -3.10291800 -0.15228800 0.03170600
C -0.30644200 4.78636000 -0.69564600
H -0.74755100 5.75698500 -0.46142100
O 1.85148300 -1.74295000 2.89513400
C -4.57860300 -0.21093800 0.14237700
O 4.89606400 -0.40293500 -0.01223900
C 3.75414500 -0.26353600 0.14221600
C -5.31080500 -1.26268600 -0.43893300
H -4.79492700 -2.03973300 -1.00570600
C 1.40234300 3.46244200 -1.80241200
H 2.29296700 3.38609600 -2.43014500
C -6.70128700 -1.30748500 -0.32916000
H -7.25397400 -2.12585000 -0.79589900
O 2.22396700 2.41873900 2.07347400
C 2.12426100 -2.90269700 -1.15213100
H 3.15133800 -2.56723600 -1.29981100
C 1.75160500 -4.21802900 -1.41487900
H 2.49136100 -4.92421300 -1.79297500
C -0.47499800 -3.65013500 -0.70686700
H -1.51291100 -3.92388000 -0.52375500
C 0.43179400 -4.60162500 -1.17116100
H 0.10759400 -5.62881800 -1.34663200
C 2.09090000 1.50215400 1.37591300
N 1.25701700 -1.98762800 -0.69037000
C 0.82195500 4.69406600 -1.52872500
H 1.25985300 5.60117600 -1.95423600
C -5.27853900 0.79184900 0.83912100
H -4.72810900 1.60424300 1.31737400
C -6.66833600 0.74260200 0.95318600
H -7.19351300 1.52427900 1.50656200
C -7.38492200 -0.30642400 0.36827400
H -8.47308500 -0.34395100 0.45593800
C 1.87024700 -1.12765200 1.90930600
H -1.74933600 3.68317200 0.44892700

1-OPh$^{\bullet-}$

-1 2
Re 1.82368900 -0.08818800 0.33142400
N -0.30507000 -0.02185000 0.02416400
C -0.05378300 -2.31403100 -0.55231500
O 1.58987500 1.13317700 -1.44093200
C -0.92878400 -1.24874000 -0.15967000
C -1.02887800 1.11963500 -0.15195900
C -0.85257200 3.61962000 -0.25431200
C -2.33283300 -1.33339000 -0.06960900
H -2.80735800 -2.31429000 -0.07462300
C 0.93702800 2.26015200 -1.24860100

```
C -0.29145100 2.35277300 -0.49978300
C -2.41854300 1.07254700 -0.13707300
H -2.98408000 1.98234600 -0.32849400
C -3.10429700 -0.17660800 0.00232100
C -0.28307000 4.79107500 -0.74837700
H -0.73501600 5.76083900 -0.52896900
O 1.79910400 -1.75678000 2.92461000
C -4.57497200 -0.22216900 0.13679100
O 4.89030100 -0.42624600 0.04860000
C 3.74298400 -0.28161300 0.19051800
C -5.32591800 -1.34788500 -0.26465500
H -4.82044100 -2.20410700 -0.71490100
C 1.46467600 3.46501100 -1.78121700
H 2.38119200 3.38782700 -2.37167400
C -6.71301500 -1.37864700 -0.12439100
H -7.26783000 -2.26197100 -0.45063500
O 2.17906400 2.42658700 2.09922100
C 2.15378300 -2.88475100 -1.16066500
H 3.18978200 -2.55205200 -1.25431800
C 1.79484800 -4.18054600 -1.48950400
H 2.54578800 -4.87804100 -1.86170300
C -0.46864400 -3.63835900 -0.85024000
H -1.51622100 -3.91246200 -0.72244000
C 0.44174300 -4.56537300 -1.31379300
H 0.11973400 -5.58210800 -1.54948200
C 2.05601600 1.50092800 1.40430000
N 1.28218900 -1.96636500 -0.69785500
C 0.87525200 4.70097400 -1.53519400
H 1.33133900 5.60721500 -1.94416700
C -5.28061400 0.87369200 0.67789900
H -4.72944500 1.75308100 1.01589300
C -6.66935200 0.84291200 0.81777500
H -7.18683700 1.70406100 1.24817300
C -7.39585900 -0.28315400 0.41830500
H -8.48275800 -0.30736000 0.52580100
C 1.82977000 -1.13484200 1.93847300
H -1.76220000 3.67941800 0.34840700
```

1-OPh^{2-}

```
-2 1
Re 1.80416800 -0.10338000 0.38297300
N -0.29273100 -0.02369300 0.08900000
C -0.05546100 -2.27697600 -0.67190700
O 1.62630200 1.15627500 -1.41103400
C -0.91960000 -1.26229600 -0.22830800
C -1.02814700 1.11120900 -0.17368100
C -0.86727500 3.61553800 -0.29337800
C -2.32672300 -1.34947900 -0.12721000
H -2.79018800 -2.33721400 -0.17062900
C 0.96055200 2.27562100 -1.23405400
C -0.29663700 2.35161300 -0.52851400
C -2.40677100 1.06224900 -0.21011800
H -2.95880800 1.95082300 -0.51142200
C -3.11170600 -0.20514500 -0.03011700
C -0.29678700 4.79801800 -0.76853000
H -0.76789900 5.76203500 -0.56097100
O 1.81679100 -1.79865100 2.95328000
C -4.56443600 -0.24993000 0.14119900
O 4.88119100 -0.40547200 0.06019500
C 3.72749000 -0.27842800 0.21341700
C -5.30340800 -1.46454000 0.14674400
H -4.78224200 -2.41544800 0.02270900
C 1.49935600 3.49075400 -1.73413900
H 2.43981900 3.42378400 -2.28854400
C -6.68593800 -1.48428500 0.30140000
H -7.21044700 -2.44429300 0.29418300
O 2.14099600 2.43121800 2.13660600
C 2.19687200 -2.83665100 -1.19533300
H 3.23946200 -2.50662900 -1.21257300
C 1.86114600 -4.10390800 -1.62590200
H 2.63068500 -4.78187800 -1.99741000
C -0.44270200 -3.59764400 -1.07759400
```

```
H -1.49843100 -3.87238000 -1.03025200
C 0.48373900 -4.49350600 -1.54640300
H 0.17210100 -5.49102000 -1.86797100
C 2.02111100 1.49229200 1.45079900
N 1.31241700 -1.93357000 -0.71603500
C 0.88938800 4.72402600 -1.51189100
H 1.35224300 5.63549100 -1.90256500
C -5.32084700 0.94061000 0.31545200
H -4.80566900 1.90131000 0.34669400
C -6.70732700 0.91690600 0.47379700
H -7.24514600 1.85997100 0.60981400
C -7.41085500 -0.29243000 0.46619800
H -8.49621700 -0.31069500 0.58964000
C 1.82860600 -1.15825300 1.97273800
H -1.79375200 3.66135000 0.28521500
```

1-PhO^{2-}-*cis*

```
-2 1
C    2.35433300   -0.96124500    0.07609100
C    3.08070500    0.27284400    0.04419200
C    2.31378300    1.41965800   -0.00825000
C    0.89880500    1.37143500   -0.03435800
C    0.97924900   -1.00512300    0.03144200
C    0.09029900    2.53260500   -0.07331000
C    0.58164900    3.86934900   -0.09417400
C   -0.27133000    4.94313500   -0.14066000
H    0.11094500    5.96552800   -0.15649000
C   -1.67912500    4.68308400   -0.16498000
C   -2.12131800    3.39051200   -0.14288500
H    2.88322000   -1.91316700    0.09319200
H   -2.40580800    5.49690900   -0.20061500
H   -3.18905300    3.17219600   -0.15885900
N    0.20720000    0.14781500   -0.02876900
N   -1.29075200    2.28715300   -0.10200400
Re  -1.94212400    0.27579200   -0.00005500
C   -2.38577800   -1.43311100   -0.79173200
O   -2.75384800   -2.41784700   -1.30009500
C   -3.77738700    0.78640200   -0.26956100
O   -4.87846200    1.15572800   -0.43899700
C   -2.31441000   -0.41825800    1.72221800
O   -2.54454900   -0.84465300    2.79151900
H    1.65925000    4.03403100   -0.07250300
H    2.79459500    2.39637700    0.00225300
C    4.55561000    0.30998000    0.08809400
C    5.28249400    1.40732100   -0.42405500
C    5.29587700   -0.75579400    0.64404200
C    6.67560200    1.44281200   -0.37118000
H    4.74756600    2.23611700   -0.89188900
C    6.69082700   -0.72129400    0.69458300
H    4.77030300   -1.61518200    1.06366500
C    7.39220400    0.37791800    0.18883300
H    7.20833100    2.30470600   -0.78142300
H    7.23409200   -1.55965700    1.13846600
H    8.48379400    0.40383700    0.22607000
C    0.33823600   -2.34713700    0.03465500
C   -0.16844600   -2.88626200    1.21764400
C    0.36516300   -3.11345400   -1.20076600
C   -0.67517900   -4.19114200    1.28412500
C   -0.12877500   -4.46356000   -1.07205700
C   -0.63765100   -4.97297500    0.11664800
H   -1.07322200   -4.59077200    2.22037600
H   -0.10896400   -5.07893000   -1.97855100
H   -1.01336800   -6.00284100    0.14265200
O    0.78919200   -2.63589000   -2.29889600
H   -0.16879100   -2.26324300    2.11732400
```

1-PhO^{2-}-*trans*

```
-2 1
C    -2.38222000    1.02545900   -0.06315200
C    -3.12788200   -0.20421600   -0.03982500
C    -2.36762100   -1.35289400    0.08025200
```

```
C    -0.95550800   -1.31963400    0.10444000
C    -1.00825500    1.05728200   -0.04249400
C    -0.16140900   -2.48572600    0.21913100
C    -0.65826800   -3.80355100    0.43827000
C     0.19341600   -4.87631700    0.52919400
H    -0.19162400   -5.88262900    0.70661300
C     1.59751300   -4.64298400    0.39702700
C     2.03942800   -3.36411900    0.18330700
H    -2.89948600    1.98354200   -0.08254500
H     2.31876600   -5.45925400    0.46410300
H     3.10458400   -3.15355100    0.07888700
N    -0.24070600   -0.11211600   -0.05500100
N     1.21167100   -2.27354600    0.08017900
Re    1.87191700   -0.26548800   -0.23612300
C     2.25840300    1.49566200   -0.96367900
O     2.60888400    2.50051000   -1.44369200
C     3.49714200   -0.88536800   -1.04340100
O     4.45570000   -1.34423000   -1.54440100
C     2.80627300    0.19669200    1.34546200
O     3.40768500    0.47857100    2.31158600
H    -1.73302600   -3.95154800    0.54741500
H    -2.85646500   -2.32127900    0.17709700
C    -4.59905700   -0.22925100   -0.09355100
C    -5.30658500   -1.39328500   -0.47596000
C    -5.36867100    0.91240700    0.22820300
C    -6.69957200   -1.41805800   -0.51986500
H    -4.75305900   -2.28820200   -0.76669100
C    -6.76360800    0.88782100    0.18016300
H    -4.86849700    1.82978500    0.54195600
C    -7.44313000   -0.27667300   -0.19247100
H    -7.21136300   -2.33465700   -0.82530200
H    -7.32551400    1.78842200    0.44212500
H    -8.53474600   -0.29460900   -0.23268500
C    -0.34188100    2.38042700    0.01205100
C    -0.48369700    3.27373600   -1.05348000
C     0.30262100    2.78664600    1.25550800
C     0.03936900    4.57240600   -1.01683000
C     0.84049800    4.12594800    1.23691600
C     0.70954700    4.98047000    0.14804800
H    -0.06512600    5.24359500   -1.87317700
H     1.34620200    4.45844300    2.15054200
H     1.13258400    5.99058500    0.20408900
O     0.35934600    2.04237300    2.28057500
H    -1.00771100    2.92756400   -1.95159200
```

1-PhO(CH_3CN)$^{2-}$-*cis*

```
-2 1
C     2.45812800    1.00691600   -0.19939900
C     3.18644900   -0.25022100   -0.11970100
C     2.40775600   -1.38516500   -0.02093300
C     0.98683500   -1.34772600   -0.01693400
C     1.07940300    1.03397100   -0.18388700
C     0.19636300   -2.50248700    0.08257600
C     0.69153600   -3.84574900    0.23303000
C    -0.15660200   -4.91514700    0.32848100
H     0.24126900   -5.92643700    0.44844000
C    -1.57705800   -4.69090800    0.27194700
C    -2.01449600   -3.39567200    0.11500900
H     2.98446100    1.95972100   -0.18810000
H    -2.29464800   -5.50905900    0.34726600
H    -3.08533100   -3.18454500    0.05933300
N     0.30504500   -0.09803900   -0.14568700
N    -1.20298800   -2.31335500    0.01730200
Re   -1.85879200   -0.27552100   -0.28506200
C    -2.41959300    1.58862000   -0.38736900
O    -2.88996600    2.65073400   -0.44943600
C    -3.72531100   -0.75219100   -0.24493800
O    -4.84646800   -1.08974300   -0.22177800
C    -1.83224200   -0.36369800   -2.19084500
O    -1.78722600   -0.40490200   -3.35570700
H     1.76962800   -4.00706500    0.28047800
H     2.88414800   -2.36388000    0.02533400
```

```
C     4.65073900   -0.30269300   -0.15913300
C     5.38405900   -1.46073900    0.21215500
C     5.41461800    0.82189800   -0.56790200
C     6.77414800   -1.49632700    0.16388000
H     4.85028800   -2.34569000    0.56419300
C     6.80963200    0.78386900   -0.61390600
H     4.90175500    1.73325000   -0.87772500
C     7.50818800   -0.37224300   -0.25042700
H     7.29735800   -2.40897500    0.46361500
H     7.35814900    1.67162300   -0.94243700
H     8.59993100   -0.39964400   -0.28223800
C     0.43226000    2.37852800   -0.20227800
C     0.15419700    3.04419200    1.05911600
C     0.23807900    3.03257800   -1.42025000
C    -0.34058400    4.39026500    0.93166700
C    -0.25670000    4.34233300   -1.49843100
C    -0.53970900    5.01084900   -0.29841100
H    -0.55330300    4.92499800    1.86431900
H    -0.41220500    4.82684300   -2.46599300
H    -0.92082700    6.03859200   -0.32589300
O     0.35377600    2.49038100    2.19198500
H     0.48236600    2.49003700   -2.34069900
C    -1.36539300    0.27775000    2.95885900
N    -1.68374200   -0.08062600    1.90712900
C    -0.94205800    0.80406200    4.24209300
H    -0.19593100    0.13737400    4.69903500
H    -0.48292800    1.78064600    4.01236400
H    -1.79980300    0.90944500    4.92220000
```

1-PhO(CH_3CN)$^{2-}$-*trans*

```
-2 1
C    -2.47731700   -0.98430700    0.08936600
C    -3.17892200    0.28424900   -0.02471000
C    -2.37870900    1.40357800   -0.11620100
C    -0.95747200    1.33552400   -0.11531800
C    -1.10096900   -1.04764400    0.02784200
C    -0.14264800    2.47574800   -0.13296100
C    -0.60849600    3.83854800   -0.11893600
C     0.26253400    4.89313900   -0.13570600
H    -0.11336100    5.91976400   -0.11978500
C     1.67834700    4.63413600   -0.16804300
C     2.08782200    3.32042200   -0.18059700
H    -3.02317700   -1.91340500    0.24322400
H     2.41324500    5.44018800   -0.17964700
H     3.15398000    3.08127000   -0.20547500
N    -0.30402000    0.06237400   -0.10810700
N     1.25325400    2.25192100   -0.16353400
Re    1.86457200    0.17858700   -0.22406500
C     2.37804700   -1.70130200   -0.14705700
O     2.82352000   -2.77522300   -0.11403900
C     3.74051000    0.61027100   -0.12872000
O     4.86809800    0.91796100   -0.06813200
C     1.97375900    0.15277700   -2.13260800
O     2.03626000    0.15425800   -3.29609800
H    -1.68276100    4.02675100   -0.08495700
H    -2.83582500    2.38736100   -0.21951400
C    -4.64308000    0.35982700   -0.05936700
C    -5.34999100    1.56952700    0.16866600
C    -5.43058700   -0.79295100   -0.31373200
C    -6.73995600    1.62529800    0.12967600
H    -4.79499900    2.48069900    0.40084600
C    -6.82517500   -0.73455500   -0.35142400
H    -4.93596600   -1.74494100   -0.51099600
C    -7.49818200    0.47176700   -0.13113500
H    -7.24366000    2.57815900    0.31619400
H    -7.39345400   -1.64587600   -0.55974300
H    -8.58958700    0.51574600   -0.15567900
C    -0.48474700   -2.40225400    0.14798300
C    -0.35526000   -3.22761000   -1.04239400
C    -0.17699000   -2.90362500    1.41381900
C     0.08137300   -4.57885400   -0.78599300
C     0.27361700   -4.21723300    1.61207100
```

```
C     0.38923600   -5.04849300    0.48692000
H     0.17628200   -5.23442300   -1.65955300
H     0.51524100   -4.58311500    2.61359700
H     0.72671500   -6.08468700    0.61033900
O    -0.61036400   -2.79942900   -2.21289300
H    -0.30192900   -2.23997500    2.27687100
C     1.32349300    0.33088000    3.07535700
N     1.60911400    0.27492300    1.95747800
C     0.94392200    0.38628000    4.47630400
H     0.18799000    1.17107700    4.62668300
H     0.52183600   -0.58203500    4.78400000
H     1.82304300    0.60949600    5.0983
```

1-PhO•CO_2^{2-}-*cis*

```
-2 1
C    2.20302800    0.79774600   -0.52441000
C    2.87530500   -0.46511500   -0.48408200
C    2.06079100   -1.57406900   -0.37613100
C    0.65269000   -1.45958200   -0.28704300
C    0.83701000    0.90656300   -0.40306300
C   -0.20010900   -2.58312100   -0.16596600
C    0.22908800   -3.94077300   -0.19191400
C   -0.66238100   -4.97448700   -0.04979700
H   -0.32787300   -6.01336400   -0.07330800
C   -2.04588400   -4.65048300    0.12398100
C   -2.42862600   -3.33862200    0.13984800
H    2.77252800    1.72305100   -0.58801900
H   -2.80077900   -5.43018300    0.24154400
H   -3.47760500   -3.07079400    0.26708600
N    0.01799200   -0.20677400   -0.26521500
N   -1.55756900   -2.27555500    0.00480000
Re  -2.12067700   -0.23444700   -0.02819500
C   -2.36062000    1.51286900    0.76814700
O   -2.59527100    2.52829700    1.29575500
C   -3.91420500   -0.65115300    0.53001500
O   -4.99024300   -0.96400100    0.87919300
C   -2.72506800    0.42243600   -1.69755100
O   -3.10268400    0.82926100   -2.73246200
H    1.28881800   -4.15504400   -0.33298600
H    2.49745800   -2.57135200   -0.37248000
C    4.34555700   -0.56263800   -0.54659900
C    5.03505800   -1.66802200   -0.00182300
C    5.11978200    0.45397300   -1.14619900
C    6.42532700   -1.75830400   -0.06485900
H    4.47320700   -2.45734100    0.50138800
C    6.51173400    0.36473800   -1.20728200
H    4.62275700    1.31809500   -1.59016700
C    7.17607300   -0.74187500   -0.66841900
H    6.92927900   -2.62433100    0.37218700
H    7.08200900    1.16589100   -1.68477900
H    8.26554900   -0.81021300   -0.71331100
C    0.26472200    2.27864500   -0.41253000
C   -0.41021300    2.74521200   -1.54330800
C    0.52247800    3.14824600    0.72194100
C   -0.87326700    4.06155400   -1.64875000
C    0.05353100    4.50180400    0.55569100
C   -0.62311900    4.93589200   -0.57709300
H   -1.40194000    4.39914600   -2.54375200
H    0.24254000    5.18720300    1.38912800
H   -0.96294500    5.97659100   -0.63458200
O    1.11287900    2.76931100    1.78607300
H   -0.57775800    2.05058700   -2.37132900
C    2.21612400    0.61301100    2.56338600
O    1.22511700    0.02037000    2.72272200
O    3.28692400    1.06991600    2.50294300
```

^{TS}PhO•CO_2^{2-}

```
-2 1
C    2.43727700    1.05452600   -0.16472400
C    3.18849700   -0.18288600   -0.09761200
C    2.43112900   -1.32008700    0.11906600
```

C	1.01870500	-1.29502700	0.17247900
C	1.07015100	1.06365800	-0.10692000
C	0.22464100	-2.43760300	0.39227000
C	0.71213400	-3.73193800	0.75111700
C	-0.14800300	-4.77756300	0.96649900
H	0.23108300	-5.76062600	1.25463500
C	-1.55509900	-4.55193300	0.82071400
C	-1.98685900	-3.30067800	0.46449400
H	2.95300100	2.01435800	-0.15229800
H	-2.28096100	-5.34934500	0.98727800
H	-3.05167400	-3.09733300	0.34040400
N	0.29965300	-0.09329200	-0.06800800
N	-1.15449300	-2.23814600	0.23648900
Re	-1.76415500	-0.33116600	-0.52241800
C	-2.32034100	1.50055100	-0.85423500
O	-2.76878400	2.55798000	-1.04607600
C	-3.64083800	-0.74746600	-0.35854000
O	-4.77086700	-1.02861100	-0.23850700
C	-1.81805200	-0.71482100	-2.35590700
O	-1.81375300	-0.98822000	-3.49786800
H	1.78671900	-3.87463600	0.87289900
H	2.92045300	-2.28898900	0.21540900
C	4.65130400	-0.20427900	-0.21656000
C	5.42812500	-1.30687900	0.21777900
C	5.35523900	0.89070100	-0.77405600
C	6.81530300	-1.31666400	0.09290400
H	4.93665900	-2.16479600	0.68044200
C	6.74588100	0.88012400	-0.89569600
H	4.80052000	1.75619100	-1.13993100
C	7.49152600	-0.22217500	-0.46508900
H	7.37921700	-2.18488500	0.44494200
H	7.25179300	1.74288800	-1.33791000
H	8.57998200	-0.22963000	-0.55839400
C	0.38048900	2.37266300	0.03210600
C	0.36357000	3.31545900	-1.00377200
C	-0.17541800	2.72034600	1.28346500
C	-0.23146700	4.56783200	-0.82740200
C	-0.78196100	3.96810100	1.45387200
C	-0.81451400	4.88815300	0.40164200
H	-0.24658600	5.28781300	-1.64922900
H	-1.20643600	4.21172200	2.43022200
H	-1.28861800	5.86236700	0.54765700
O	-0.01053700	1.87609700	2.34069000
H	0.81359700	3.04811800	-1.96306500
C	-1.07594900	0.96578600	2.72963700
O	-2.15907500	1.13092800	2.14673200
O	-0.71149500	0.16643600	3.59049500

1-OCOOPh^{2-}

-2 1

C	-2.45814600	-1.12443300	-0.03851300
C	-3.20390500	0.13116700	-0.05794600
C	-2.43959500	1.27636200	0.11043800
C	-1.02865600	1.25165300	0.18601100
C	-1.08889200	-1.11950500	0.01343300
C	-0.22469900	2.38332000	0.41296100
C	-0.69854500	3.69487600	0.74239100
C	0.17423900	4.72374900	0.98489100
H	-0.20058900	5.71483500	1.25310500
C	1.58426400	4.47870500	0.89753700
C	1.99980500	3.21300800	0.55193200
H	-2.97448100	-2.07840000	0.06331200
H	2.31697300	5.26364300	1.08972100
H	3.06550600	2.99041800	0.45698200
N	-0.32586800	0.02776000	-0.02405100
N	1.16280000	2.17492700	0.29674000
Re	1.77015800	0.23611100	-0.52361900
C	2.23778600	-1.53894600	-1.14490500
O	2.58870900	-2.57718700	-1.54220800
C	3.64823100	0.68044000	-0.61128300
O	4.77591800	0.98832200	-0.65023900
C	1.52484400	0.82129600	-2.29700000

O	1.33990400	1.20397700	-3.38823600
H	-1.77411900	3.86015600	0.82531500
H	-2.92695500	2.25024500	0.16546500
C	-4.65830500	0.15729900	-0.20546500
C	-5.43383900	1.32249200	0.04121100
C	-5.37660100	-1.00093900	-0.60604500
C	-6.81723000	1.32837300	-0.10646900
H	-4.94167600	2.23950700	0.37050700
C	-6.76452500	-0.99110100	-0.75284100
H	-4.83182100	-1.91936600	-0.82885300
C	-7.50450400	0.17028900	-0.50541600
H	-7.37278400	2.24789500	0.09943700
H	-7.27438500	-1.90548800	-1.07030500
H	-8.59111400	0.17675200	-0.61821400
C	-0.36144900	-2.39683200	0.22712200
C	-0.40822200	-3.44437300	-0.70223800
C	0.35694800	-2.59328900	1.42755500
C	0.27055600	-4.64503800	-0.47464300
C	1.04798500	-3.78542200	1.65006500
C	1.01135100	-4.81035500	0.69769100
H	0.23138900	-5.44449900	-1.21845300
H	1.58886300	-3.90745200	2.59115500
H	1.55423300	-5.74104000	0.88097300
O	0.25637300	-1.67559400	2.44333300
H	-0.97350300	-3.29579100	-1.62529900
C	1.13640600	-0.57121300	2.57053900
O	0.87447600	0.16366800	3.51130900
O	2.08115600	-0.50919200	1.73375200
